# Involvement of propionate, citrulline, homoserine, and succinate in oral microbiome metabolite-driven periodontal disease progression

**DOI:** 10.1038/s41598-025-91105-w

**Published:** 2025-02-28

**Authors:** Chikako Ishihara, Misato Sako, Kota Tsutsumi, Narumi Fujii, Daiki Hashimoto, Atsushi Sato, Yuko Ichiba, Takashi Chikazawa, Yasushi Kakizawa, Eiji Nishinaga, Akira Uchiyama

**Affiliations:** 1 Research and Development Headquarters, Lion Corporation, 7-2-1 Hirai, Edogawa-ku, Tokyo, 132-0035 Japan; 2 Section of Oral Health Promotion and Technology, Division of Oral Health, Technology and Epidemiology, Kyushu University Faculty of Dental Science, Fukuoka, Fukuoka 812-8582 Japan; 3 The Lion Foundation for Dental Health, 1-3-28 Kuramae, Taito-ku, Tokyo, 111-8644 Japan

**Keywords:** Oral Microbiome, Metabolomics, Periodontal desease, Microbiology, Pathogenesis

## Abstract

Dysbiosis of the oral microbiome has been implicated in the onset and progression of periodontal diseases. An altered oral microbiome can significantly affect the concentration and composition ratio of bacterial-derived metabolites, thereby contributing to disease development. However, there is limited research on the role of metabolites derived from the oral microbiota. This study aimed to identify specific bacteria-derived metabolites and their contributions to pathogenicity. Mouth-rinsed water was collected from 24 patients with periodontal disease and 22 healthy individuals. We conducted a correlation analysis between periodontal disease-associated bacteria and metabolites present in mouth-rinsed water. We evaluated the effects of these metabolites on human gingival epithelial cells analysis of oral bacteria culture supernatants confirmed the origin of these metabolites. We identified 20 metabolites associated with bacteria that are significantly more prevalent in periodontal disease. Notably, propionate, succinate, citrulline, and homoserine—metabolites derived from the oral microbiome—were identified as being associated with periodontal disease. These results suggested that metabolites derived from the oral microbiota are involved in periodontal disease.

**Supplementary Information:**

The online version contains supplementary material available at 10.1038/s41598-025-91105-w.

## Introduction

Periodontal disease is a major global health concern and a prominent issue in oral health^1^. Dysbiosis of the oral microbiome is a significant contributing factor in the development and progression of periodontal diseases^2^. Dysbiosis of the oral microbiome affects the quantity and compositional balance of metabolites produced^3,4^. However, limited studies have comprehensively linked the oral microbiome with its metabolites^5,6^. Understanding the association between oral microbiome and its corresponding metabolites can provide important insights into the complex mechanisms underlying the onset and progression of periodontal diseases, leading to more effective therapeutic interventions.

In recent years, studies on oral cavity metabolites have made notable progress^7^. However, most of this research has focused on the exploration of biomarkers^8–13^, and their role in the onset and progression of periodontal disease remains unclear. The potential involvement of metabolites derived from the oral microbiome in the pathogenesis of periodontal disease should be further investigated. Therefore, we propose the hypothesis that changes in metabolites caused by oral microbiome dysbiosis may contribute to the onset and progression of periodontal disease.

This study aimed to identify metabolites derived from the oral microbiota that are involved in periodontal disease. Using an integrative approach, we combined correlation analysis of the oral microbiome and metabolites in mouth-rinsed water with in vitro analysis of their effects on human gingival epithelial cells and metabolite production by the oral microbiome. Through this approach, we investigated the association between oral microbiome-derived metabolites and periodontal disease. Studying the oral microbiome and its metabolites can enhance our understanding of periodontal disease.

## Results

### Metabolites in mouth-rinsed water correlated with bacteria significantly more prevalent in the periodontal disease group

**Fig. 1:**
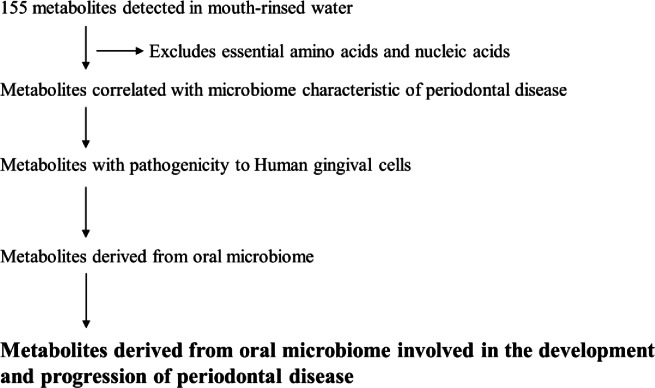
Flow diagram of metabolites involved in the development and progression of periodontal disease.

To identify bacteria-derived metabolites involved in periodontal disease, we collected mouth-rinsed water from 22 healthy individuals and 24 patients with periodontal^14^. The healthy and periodontal disease groups were defined based on oral health indices [number of teeth, bleeding on probing (BOP), and a probing pocket depth (PPD) of 4 mm or more (PPD ≥ 4 mm)] (Table 1). The periodontal disease group exhibited significantly higher values for maximum PPD, the number of BOP sites, the number of teeth exhibiting BOP, and the percentage of BOP compared to the healthy group. The Wilcoxon rank-sum test and linear discriminant analysis effect size (LefSE) indicated 24 bacterial species with significantly higher abundance ratios in the mouth-rinsed water of the periodontal disease group (Table 2, Supplementary Table S1). Among these, *Porphyromonas gingivalis*, *Treponema denticola*, *Tannerella forsythia*, *Fusobacterium nucleotum* subsp. *vincentii*, and *Veionella parvula* are known to be associated with periodontal disease^15^. *P. gingivalis* and *F. nucleotum* subsp. *vincentii* have been shown to be associated with inflammation, but the role of the other microbes is unclear^16^. Furthermore, we performed a correlation analysis between these 24 bacterial species and the 155 metabolites detected in the mouth-rinsed water (Fig. 1). As a result, 31 metabolites with a correlation coefficient of 0.4 or higher were identified. Excluding essential amino and nucleic acids, 20 components were identified as metabolites that may be involved in periodontal disease (Table 3, Supplementary Table S2).

**Table 1:** Clinical condition of each study participant.

	Group	
	Healthy	Periodontal disease
Number of participants (female/male)	22 (12/10)	24 (9/15)
Age (years)	43.68 (± 11.82)	49.33 (± 12.03)
Number of teeth	27.95 (± 1.09)	27.46 (± 3.34)
Average PPD (mm)	2.61 (± 0.27)	2.91 (± 0.90)
Max PPD (mm)	3.00 (± 0.00)	6.46 (± 3.08)*
Number of BOP sites	2.45 (± 2.34)	39.21 (± 25.78)*
Number of teeth with BOP	1.82 (± 1.76)	14.21 (± 6.57)*
Rate of BOP (%)	2.19 (± 2.09)	36.02 (± 22.44)*

Data are shown as number (%) or median, and the means ± SDs were calculated. Comparison between the healthy group and periodontal disease group: Wilcoxon’s rank-sum test.

*P < 0.05.

**Table 2:** List of bacteria that were significantly more prevalent in the periodontal disease group.

species	Health_max	Health_median	Health_ave	Perio_max	Perio_median	Perio_ave	LDA score
*Prevotella oris*	1.30	0.01	0.09	1.16	0.15**	0.28	2.90*
*Prevotella denticola*	0.03	0.00	0.00	0.20	0.03**	0.05	2.38*
*Tannerella forsythia*	0.03	0.00	0.00	0.54	0.02**	0.08	2.61*
*Prevotella veroralis*	0.07	0.00	0.00	0.34	0.02**	0.07	2.47*
*Parvimonas micra*	0.17	0.00	0.02	0.46	0.03**	0.09	2.60*
*Peptostreptococcaceae [XI][G-6] [Eubacterium] nodatum*	0.00	0.00	0.00	0.20	0.00**	0.03	2.23*
*Dialister invisus*	0.20	0.01	0.02	0.19	0.04**	0.05	2.36*
*Fusobacterium nucleatum* subsp. *vincentii*	1.34	0.04	0.13	4.73	0.21**	0.48	3.26*
*Stomatobaculum longum*	0.08	0.00	0.01	0.44	0.01**	0.05	2.33*
*Streptococcus parasanguinis clade 411*	1.36	0.07	0.25	4.88	0.82**	1.33	3.71*
*Treponema socranskii*	0.03	0.00	0.00	0.40	0.02**	0.04	2.30*
*Porphyromonas gingivalis*	0.05	0.00	0.00	5.23	0.04**	0.55	3.48*
*Fretibacterium fastidiosum*	0.00	0.00	0.00	0.18	0.00**	0.01	2.24*
*Shuttleworthia satelles*	0.00	0.00	0.00	0.17	0.00**	0.01	2.16*
*Atopobium rimae*	0.06	0.00	0.00	0.13	0.01**	0.02	2.18*
*Dialister pneumosintes*	0.10	0.00	0.01	0.14	0.01**	0.02	2.18*
*Veillonella parvula*	2.71	0.22	0.47	11.13	1.19**	1.73	3.73*
*Atopobium parvulum*	0.11	0.02	0.02	0.39	0.05**	0.06	2.36*
*Prevotella dentalis*	0.00	0.00	0.00	0.05	0.00**	0.01	2.10*
*Pseudoramibacter alactolyticus*	0.00	0.00	0.00	0.20	0.00**	0.02	2.06*
*Treponema* sp. *HMT 237*	0.01	0.00	0.00	0.38	0.00**	0.05	2.40*
*Filifactor alocis*	0.13	0.00	0.01	2.24	0.01**	0.15	2.94*
*Porphyromonas endodontalis*	0.59	0.00	0.06	1.93	0.05**	0.24	3.01*
*Treponema denticola*	0.05	0.00	0.01	2.55	0.01**	0.18	2.99*

**Comparison between the healthy group and periodontal disease group: Wilcoxon’s rank-sum test, *P* < 0.01.

*LDA score > 2 and *P* < 0.05, as determined by linear discriminant analysis effect size (LEfSe) analysis.

**Table 3:** List of metabolites correlated with bacteria significantly abundant in the mouth-rinsed water of the periodontal disease group.

Metabolite	Category	Correlation coefficient	Concentration in mouth-rinsed water (µM)
Taurine	Amino acid derivative	0.60*	34.74
*N*-Gamma-ethylglutamine	Amino acid derivative	0.57*	0.16
5-Oxoproline	Amino acid derivative	0.56*	2.15
*N*-Gamma-ethylglutamine	Amino acid derivative	0.56*	0.16
2-Hydroxy-4-methylpentanoate	Short-chain fatty acid	0.51*	0.35
Ala-Ala	Dipeptide	0.50*	0.15
Citrulline	Amino acid derivative	0.49*	3.98
*N*1, *N*12-Diacetylspermine	Polyamine	0.49*	0.03
Propionate	Short-chain fatty acid	0.48*	44.89
UDP-glucose	Sugar metabolite	0.48*	0.06
2-Aminobutyrate	Amino acid derivative	0.48*	0.30
Succinate	Organic acid	0.45*	15.59
2-Hydroxypentanoate	Short-chain fatty acid	0.44*	0.51
Pyruvate	Organic acid	0.43*	16.54
2-Hydroxyglutarate	Short-chain fatty acid	0.43*	1.41
Citramalate	Organic acid	0.43*	1.41
Trehalose 6-phosphate	Sugar metabolite	0.42*	0.09
Urocanate	Amino acid derivative	0.42*	0.47
Glucose-6-phosphate	Sugar metabolite	0.41*	2.08
Homoserine	Amino acid derivative	0.41*	0.10
α-Aminoadipate	Amino acid derivative	0.41*	0.50

Categories indicate the category to which the metabolite belongs, and correlations indicate values from Spearman analysis. Asterisks indicate significant test results. The concentrations of each metabolite in mouth-rinsed water are listed. Spearman |ρ| ≥ 0.4. Significant differences were calculated using the Bonferroni method. Bonferroni method *P* < 0.05. Excluding essential amino and nucleic acids.

### Some metabolites correlated with predominant bacteria in the periodontal disease group exhibit cell growth-inhibitory and inflammation-inducing effects

To assess growth inhibition and inflammatory cytokine expression as indicators of tissue inflammation and destruction in key pathological conditions of periodontal disease^17^, we evaluated the effects of 20 metabolites (Table 3) that are considered to be correlated with bacteria in human gingival epithelial cells. The *N*1, *N*12-diacetylspermine treatment group showed significantly inhibited epi4 cell growth compared with that of the control group. In addition, treatment with citrulline or succinate inhibited epi4 cell growth (Table 4). Treatment with 5-oxoproline, homoserine, propionate, and succinate significantly increased IL-8 mRNA expression compared with that in the control group, and treatment with *N*1, *N*12-diacetylspermine showed a trend of increasing IL-8 mRNA expression (*P* = 0.07) (Table 5).

**Table 4:** Growth-inhibitory effects of metabolites significantly correlated with bacteria present in the mouth-rinsed water of the periodontal disease group on human gingival epithelial cells.

Metabolite	Growth-inhibitory effects vs. control		
	Metabolite concentration		
	×1000 (×100^a^)	×100 (×10^a^)	Saliva
Taurine	0.99 ± 0.06	1.05 ± 0.06	1.02 ± 0.03
*N*-Gamma-ethylglutamine	1.10 ± 0.02	1.03 ± 0.03	1.01 ± 0.06
5-Oxoproline	1.15 ± 0.11	1.21 ± 0.04*	0.96 ± 0.03
2-Hydroxy-4-methylpentanoate	1.02 ± 0.05	1.07 ± 0.02	1.13 ± 0.06^†^
Ala-Ala	1.19 ± 0.11^†^	1.14 ± 0.04	1.23 ± 0.04*
Citrulline	0.90 ± 0.05	1.03 ± 0.08	0.85 ± 0.13^†^
*N*1, *N*12-Diacetylspermine	0.62 ± 0.02***	0.64 ± 0.06***	0.65 ± 0.05***
Propionate^a^	1.13 ± 0.07^†^	1.14 ± 0.12	0.90 ± 0.08
UDP-glucose	1.06 ± 0.10	1.10 ± 0.04	1.03 ± 0.07
2-Aminobutyrate	1.08 ± 0.03	1.15 ± 0.14	1.09 ± 0.14
Succinate	1.05 ± 0.11	1.20 ± 0.04*	0.83 ± 0.13^†^
2-Hydroxypentanoate	1.16 ± 0.04	1.11 ± 0.05	1.31 ± 0.26
Pyruvate	1.19 ± 0.11	1.08 ± 0.07	0.89 ± 0.11
2-Hydroxyglutarate	1.04 ± 0.14	0.97 ± 0.12	1.01 ± 0.06
Trehalose 6-phosphate	1.09 ± 0.03	1.14 ± 0.04*	1.05 ± 0.05
Urocanate	1.11 ± 0.06	1.10 ± 0.03	1.00 ± 0.08
Glucose-6-phosphate	1.16 ± 0.08	1.08 ± 0.04	1.01 ± 0.04
Homoserine	1.15 ± 0.04*	1.0 ± 0.10	1.03 ± 0.03
α-Aminoadipate	0.89 ± 0.04	0.86 ± 0.05	0.99 ± 0.16

The study was conducted with *n* = 3–6, and the means ± SDs were calculated. Dunnett’s test was used to calculate significant differences between the control and metabolite addition groups. ***P < 0.001, **P < 0.01, *P < 0.05, ^†^*P* < 0.1.

×1000 means a concentration in saliva calculated to be 1000 times the average concentration found in the mouth-rinsed water of the periodontal disease group.

×100 means a concentration in saliva calculated to be 100 times the average concentration found in the mouth-rinsed water of the periodontal disease group.

×10 means a concentration in saliva calculated to be 10 times the average concentration found in the mouth-rinsed water of the periodontal disease group.

^a^Maximum concentration in saliva ×100.

**Table 5:** Inflammation-promoting effect of metabolites correlated with bacteria that were significantly more prevalent in the periodontal disease group on human gingival epithelial cells.

Metabolite	IL-8 expression vs. control		
	Metabolite concentration		
	×1000 (×100^a^)	×100 (×10^a^)	Saliva
Taurine	0.66 ± 0.25	0.66 ± 0.03*	0.65 ± 0.08*
*N*-Gamma-ethylglutamine	1.35 ± 0.68	1.50 ± 0.54	1.12 ± 0.52
5-Oxoproline	3.11 ± 1.79*	3.31 ± 1.37*	2.15 ± 1.05
2-Hydroxy-4-methylpentanoate	0.64 ± 0.11***	0.62 ± 0.06***	0.64 ± 0.06***
Ala-Ala	0.75 ± 0.05***	0.57 ± 0.14***	0.56 ± 0.10***
Citrulline	0.96 ± 0.23	0.80 ± 0.10	0.82 ± 0.19
*N*1, *N*12-Diacetylspermine	1.64 ± 0.14^†^	1.31 ± 0.33	1.18 ± 0.09
Propionate^a^	2.67 ± 0.31***	1.96 ± 0.18***	1.40 ± 0.18**
UDP-glucose	0.75 ± 0.08**	0.63 ± 0.06***	0.65 ± 0.09***
2-Aminobutyrate	1.23 ± 0.52	0.93 ± 0.39	1.34 ± 0.19
Succinate	17.62 ± 6.61***	6.65 ± 3.02**	5.37 ± 2.24^†^
2-Hydroxypentanoate	1.31 ± 0.11	1.09 ± 0.54	0.82 ± 0.14
Pyruvate	0.48 ± 0.09***	0.55 ± 0.09***	0.50 ± 0.03***
2-Hydroxyglutarate	0.8 ± 0.10	1.02 ± 0.19	1.02 ± 0.15
Trehalose 6-phosphate	0.87 ± 0.06	1.20 ± 0.52	0.70 ± 0.15
Urocanate	0.67 ± 0.21*	0.39 ± 0.16***	0.43 ± 0.18***
Glucose-6-phosphate	1.14 ± 0.08	1.30 ± 0.17	0.87 ± 0.21
Homoserine	1.58 ± 0.44	2.82 ± 1.09***	0.93 ± 0.14
α-Aminoadipate	1.23 ± 0.10	1.43 ± 0.35	0.95 ± 0.15

The study was conducted with *n* = 4–6, and the means ± SDs were calculated. Dunnett’s test was used to calculate significant differences between the control and metabolite addition groups. ***P < 0.001, **P < 0.01, *P < 0.05, ^†^*P* < 0.1.

×1000 means a concentration in saliva calculated to be 1000 times the average concentration found in the mouth-rinsed water of the periodontal disease group.

×100 means a concentration in saliva calculated to be 100 times the average concentration found in the mouth-rinsed water of the periodontal disease group.

×10 means a concentration in saliva calculated to be 10 times the average concentration found in the mouth-rinsed water of the periodontal disease group.

^a^Maximum concentration in saliva ×100.

### Metabolites with cell growth-inhibitory and inflammation-inducing effects on human gingival epithelial cells are produced by bacteria forming the oral Microbiome

Metabolites with cell growth-inhibitory and inflammation-inducing effects on human gingival epithelial cells are produced by bacteria forming the oral microbiome. To determine whether oral bacteria produce metabolites with these effects on human gingival epithelial cells, we measured the levels of metabolites in the culture supernatants of *Prevotella melaninogenica*, *Porphyromonas gingivalis* (ATCC33277), *Prevotella intermedia* (ATCC49011), *Fusobacterium nucleatum vincentii* (ATCC49256), and *Fusobacterium nucleatum nucleatum* (ATCC23726), which are common oral bacteria^18^. As shown in Table 6, propionate was detected in the culture supernatants of all tested bacteria. Succinate was detected in the culture supernatants of *P. melaninogenica*, *P. intermedia*, and *F. nucleatum nucleatum*. Homoserine was detected in the culture supernatants of *P. melaninogenica*, *P. intermedia*, and *P. gingivalis*. Citrulline was only detected in the culture supernatant of *P. gingivalis*, and 5-oxoproline and *N*1, *N*12-diacetylspermine were not detected in any of the bacteria (Table 6).

**Table 6:** Production level of each metabolite from oral bacteria.

		Metabolite (µM)					
		Propionate	5-Oxoproline	Homoserine	*N*1,*N*12-Diacetylsperspermine	Citrulline	Succinate
Species	*Prevotella melaninogenica*	47.5	N.D	0.62	N.D	N.D	1363.6
	*Prevotella intermedia*	52.1	N.D	1.12	N.D	N.D	2503.6
	*Fusobacterium nucleatum* subsp. *vincentii*	431.5	N.D	N.D	N.D	N.D	N.D
	*Fusobacterium nucleatum nucleatum*	447.5	N.D	N.D	N.D	N.D	4.0
	*Veillonella parvula*	3414.4	N.D	N.D	N.D	16.6	N.D
	*Porphyromonas gingivalis*	285.3	N.D	1.12	N.D	460.8	N.D

The study was conducted with *n* = 3.

*N.D* not detected.

## Discussion

In recent years, emphasis on the correlation between oral microbiota and their metabolites in periodontal disease studies has increased^7,19^. We hypothesized that investigating these metabolites may elucidate their potential roles in microbial pathogenicity. This study aimed to identify bacteria-derived metabolic products involved in periodontal disease. As a result, 20 metabolites were identified as metabolites correlated with characteristic bacterial groups in the periodontal disease group (Table 3). Notably, propionate, citrulline, homoserine, and succinate metabolites derived from the oral microbiota were found to play roles in cell growth inhibition and inflammation.

The present study correlated the bacterial characteristics of periodontal disease groups with metabolites detected in mouth-rinsed water. Overall, 20 metabolites were identified, including amino acid derivatives, short-chain fatty acids, sugar metabolites, dipeptides, polyamines, and organic acids (Table 3). Although previous studies have reported the presence of urocanate, 2-hydroxy-4-methylpentanoate, 5-oxoproline, taurine, citrulline, propionate, pyruvate, succinate, and glucose-6-phosphate^9,12,14,20,21^, we have newly identified 11 other metabolites. Through our targeted correlation analysis between metabolites and the 24 principal bacterial groups implicated in both periodontal disease and microbiome dysbiosis, we identified not only previously recognized metabolites but also novel ones associated with these bacteria.

In this study, citrulline, *N*1, *N*12-diacetylspermine, 5-oxoproline, homoserine, propionate, and succinate were found to exhibit cell growth-inhibitory and inflammation-inducing effects in human oral epithelial cells (Tables 4 and 5). Propionate, succinate, and 5-oxoproline are reportedly associated with periodontal disease^9,20,22,23^, and propionate and succinate have been reported to be pathogenic to human gingival epithelial cells^23–25^. In the current study, adding propionate and succinate to human oral epithelial cells elevated IL-8 gene expression (Table 5). Short-chain fatty acids exhibit antimicrobial properties in the gut, but previous reports have suggested that they function differently in the oral cavity^26,27^. Citrulline is a free amino acid, and according to Balci et al.^28^, it is significantly increased in the saliva of patients with periodontal disease, and *P. gingivalis* harbors a citrulline-producing enzyme^28,29^. As a non-protein amino acid, homoserine is a known precursor of various biologically active substances^30^, but few detailed reports on its biological activity are available. *N*1, *N*12-diacetylspermine is commonly detected in the urine of patients with breast or colorectal cancer and has been reported to be a potential biomarker^31^. The pathogenicity of 5-oxoproline, citrulline, homoserine, and *N*1, *N*12-diacetylspermine in human gingival epithelial cells has not been previously investigated. Notably, their cell growth-inhibitory and inflammation-inducing effects on human gingival epithelial cells were confirmed in the present study. These findings indicate that several metabolites are involved in periodontal diseases.

Metabolites in the oral cavity include those derived not only from the oral microbiome but also from human gingival epithelial cells. In the current study, propionate, homoserine, citrulline, and succinate were produced by periodontal disease-associated bacteria (Table 6). Propionate, citrulline, and succinate are reportedly produced by periodontal disease bacteria represented by *P. gingivaris*^24,26,29^, consistent with the results of our present study. Homoserine is known to be mainly produced by *Escherichia coli*, and Liu et al. demonstrated the activation of the homoserine degradation pathway of bacteria in the saliva of healthy individuals^32,33^. However, no reports have detected its production by periodontal disease bacteria; thus, this is a new finding. The production of 5-oxoproline and *N*1, *N*12-diacetylspermine has previously been detected in various human cells^34,35^. The 5-oxoproline and *N*1, *N*12-diacetylspermine detected in mouth-rinsed water in the present study are believed to be derived from human cells. Metabolites in mouth-rinsed water, produced by periodontal bacteria exhibit cell growth-inhibitory and inflammation-inducing effects on human gingival epithelial cells, suggesting the involvement of oral microbiome-derived metabolites in periodontal diseases.

Our study has some limitations. First, it was conducted at a single facility, necessitating further validation in a larger population. Second, this study utilized mouth-rinsed water as the sample. Recent studies have reported that analyzing the salivary microbiota can predict the state of microbial communities in dental plaque^36^. Additionally, several reports have compared disease and healthy groups using salivary microbiota samples^37^. Furthermore, it has been demonstrated that the microbes and metabolites in mouth-rinsed water reflect the information present in saliva^38,39^, thereby validating the use of mouth-rinsed water as an appropriate sample, as adopted in the present study. However, in periodontal research, it is well established that plaque samples from within periodontal pockets more accurately reflect the microbiota at lesion sites, highlighting the importance of analyzing plaque samples. Additionally, understanding the complex molecular mechanisms underlying periodontal disease requires the exploration of its relationship with lipopolysaccharides (LPS), gingipains, and microbiome-derived pathogens. Further analysis of the interactions between these pathogens could contribute to elucidating the onset and progression mechanisms of periodontal disease.

In this study, we analyzed bacteria associated with periodontal disease and determined that in addition to propionate and succinate, citrulline and homoserine produced by oral bacteria exhibit cell growth-inhibitory and inflammation-inducing effects on human gingival epithelial cells associated with periodontal disease. These findings suggest that the metabolites produced by the microbiome may contribute to periodontal disease. By conducting thorough interaction analyses with well-known pathogenic factors of periodontal disease, such as LPS, we can gain a deeper understanding of the intricate molecular mechanisms underpinning periodontal disease.

## Methods

### Study design

This experiment was conducted as described by Yama et al.^14^ between February 2018 and January 2020. The protocol for this study was approved by the Institutional Review Board of the Chiyoda Paramedical Care Clinic (Chiyoda, Tokyo; Issue number: UMIN000031334) and was conducted in accordance with the Helsinki Declaration of 1975, revised in 2013. Participants were recruited from patients who visited Hiyoshi Oral Health Clinics in Sakata City. Informed consent was obtained from the participants. In the recruited participants, the PPD at four locations per tooth and the presence or absence of BOP were confirmed through an oral examination. Exclusion criteria were as described previously^14^. The final decision was made by a dentist. Trained dental hygienists working in the same dental office performed the dental examinations. The healthy group comprised 22 individuals without a periodontal pocket of 4 mm or more and a history of caries diagnosis for 5 years or more. The periodontal disease group comprised 24 patients without caries, with at least one periodontal pocket of 4 mm or more, and with bleeding from that periodontal pocket.

### Collection of mouth-rinsed water

The mouth was vigorously rinsed for 10 s with 3 mL of sterile water, and the water was used as the saliva sample. Mouth-rinsed water was collected by a dentist or dental hygienist. Eating, drinking, or oral cleaning other than with water was stopped 1 h before oral samples were collected. After sample collection, the samples were immediately stored at − 80 °C and transported to the Lion Corporation laboratory within 3 months. After transportation, the samples were thawed at 4 °C and centrifuged at 4 °C and 13,000 × *g* for 5 min. After centrifugation, the pellets and supernatants were separately stored at − 80 °C, and the pellets were subjected to microbiome analysis, whereas the supernatants were subjected to metabolite analysis.

### Analysis of metabolites in mouth-rinsed water

Metabolites in mouth-rinsed water were analyzed according to the sample treatment methods and CE-TOF/MS analysis described in our previous study^14^. CE-TOF/MS analysis was performed using an Agilent 7100 Capillary Electrophoresis System (Agilent Technologies Deutschland GmbH, Waldbronn, Germany), Agilent 6224 TOF LC/MS System, Agilent 1200 Series Isocratic HPLC Pump, G1603A Agilent CE-MS Adapter Kit, and G1607A Agilent CE-Electrospray Ionization-MS Sprayer Kit (Agilent Technologies, Santa Clara, CA, USA), and data processing was performed using the metabolome analysis software MasterHands (Agilent Technologies)^40^. Batch-to-batch variations in measurements were adjusted using quality control (QC) samples prepared by mixing the supernatant samples from multiple participants in each group. For each measurement, the concentration of each subject’s sample was adjusted based on the concentration of the 155 types of metabolites detected in the QC samples.

### Analysis of bacteria in mouth-rinsed water

Bacteria in mouth-rinsed water were analyzed according to the sample treatment methods and NGS measurements described in our previous study^14^. The base sequences of the DNA libraries were determined using a MiSeq Sequencer (Illumina, San Diego, CA, USA). Reads with a mean quality value lower than 25 and those without primer sequences at either end were excluded. After trimming the primer sequences at both ends of the reads that passed through the filter, 10,000 reads per sample were randomly selected and grouped into operational taxonomic units (OTUs) using the UCLUST algorithm (v.5.2.32) with an identity threshold of 97%. Data from all the recruited participants, including those whose AT data had not been collected, were used for OTU grouping. The taxonomic assignment of each OTU was performed by a similarity search of the HOMD 16 S rRNA RefSeq database (version 15.22) using the GLSEARCH program (v.36.3.8 g). A sequence similarity threshold of 99% was applied for assignment to the species level.

### Extraction of metabolites correlated with bacterial groups prevalent in the periodontal disease group

Bacteria that were significantly more prevalent in the periodontal disease group were identified using the Wilcoxon rank-sum test and LEfSe analysis^41^. To extract metabolites more strongly associated with periodontal disease, significantly more prevalent bacteria in the periodontal disease group (*P* < 0.001) were selected. The correlation between this bacterial group and its metabolites was investigated using Spearman analysis. A correlation coefficient of 0.4 or higher was considered indicative of correlation, and essential amino and nucleic acids were excluded.

### Evaluation of pathogenicity in human gingival epithelial cells

#### Reagent information

In vitro tests were performed using taurine (Nacalai Tesque Inc., Kyoto, Japan), 5-oxoproline, *N*-gamma-ethylglutamine, and homoserine (FUJIFILM Wako Pure Chemical Corporation, Osaka, Japan), 2-hydroxy-4-methylpentanoate (BACHEM AG, Bubendorf, Switzerland); UDP-glucose (MedChemExpress, Monmouth Junction, NJ, USA); Ala-Ala, pyruvate, trehalose 6-phosphate, urocanate, and α-aminoadipate (Sigma-Aldrich, St. Louis, MO, USA); citrulline, propionate, and succinate (Kanto Chemicals Co., Inc., Tokyo, Japan); *N*1, *N*12-diacetylspermine (Cayman Chemical, Ann Arbor, MI, USA); 2-aminobutyrate (Tokyo Chemical Industry Co., Ltd., Tokyo, Japan); 2-hydroxypentanoate (Combi-Blocks Inc., San Diego, CA, USA); 2-hydroxyglutarate (Sigma-Aldrich); glucose-6-phosphate (Oriental Yeast Co., Ltd., Tokyo, Japan).

#### Cell culture

The human oral epithelial cell line epi4 (Osaka University Graduate School of Dentistry, Osaka, Japan) was cultured according to the method described by Yutori et al. using HuMedia-KG2 (Kurabo Industries Ltd., Osaka, Japan)^42,43^.

#### Preparation and concentration setting of evaluation substances

The evaluation substances were dissolved with the solvents shown in Table 7 at 100-fold the maximum addition concentration, added to the medium to achieve a final concentration of 1%, filtered (water-soluble: Millex-GV Syringe Filter Unit, 0.22 μm, PVDF, 33 mm, gamma sterilized SLGVR33RS; water insoluble: [Millex]-LG, 0.20 μm, hydrophilic, PTFE, 25 mm, ethylene oxide sterilized SLLG025SS; MilliporeSigma, Burlington, MA, USA), and then added to the cells. In this study, the concentration in saliva was calculated from the concentration of metabolites detected in mouth-rinsed water, and the evaluation was performed at three concentrations: estimated concentration in saliva^38^, 100 or 10 times the concentration in saliva, and 1,000 (assuming the concentration in gingival crevicular fluid^44^) or 100 times the concentration in saliva^22^ (Table 7). A stock solution equivalent to 100 times the maximum evaluated concentration was prepared and added to the cells at a final concentration of 1%. α-Aminoadipate and propionate were evaluated at saliva concentration, 10 times the saliva concentration, and 100 times the saliva concentration, whereas the other components were evaluated at saliva concentration, 100 times the saliva concentration, and 1000 times the saliva concentration.

**Table 7:** Cell evaluation concentration and solvent of each metabolite.

Metabolite	Solvent	Concentration in mouth-rinsed water (µM)	Concentration in saliva (µM)	Maximum scale (of saliva concentration)
Taurine	Water	34.74	121.59	1000
*N*-Gamma-ethylglutamine	Water	0.16	0.55	1000
5-Oxoproline	Water	2.15	7.54	1000
2-Hydroxy-4-methylpentanoate	Water	0.35	1.23	1000
Ala-Ala	Water	0.15	0.53	1000
Citrulline	Water	3.98	11.29	1000
*N*1, *N*12-Diacetylspermine	DMSO	0.03	0.12	1000
Propionate	Water	44.89	157.10	100
UDP-glucose	Water	0.06	0.20	1000
2-Aminobutyrate	Water	0.30	0.99	1000
Succinate	Water	15.59	54.56	1000
2-Hydroxypentanoate	Water	0.51	1.79	1000
Pyruvate	Water	16.54	57.88	1000
2-Hydroxyglutarate	Water	1.41	4.92	1000
Trehalose 6-phosphate	Water	0.09	0.33	1000
Urocanate	Water	0.47	1.65	1000
Glucose-6-phosphate	Water	2.08	7.11	1000
Homoserine	Water	0.10	0.35	1000
α-Aminoadipate	Water	0.50	1.74	100

#### Cell growth evaluation test

A WST-1 assay was used to test cytotoxicity. WST-1 Cell Proliferation Reagent (11644807001; Roche, Basel, Switzerland) was used according to the manufacturer’s instructions. Briefly, epi4 was seeded into 96-well plates (SUMILON MS-8096 F 96-well plate; Sumitomo Bakelite Co., Ltd., Tokyo, Japan) at 0.9 × 10^4^ cells/well, and pathogenic metabolites were added at various concentrations 3 days later. Thereafter, dimethyl sulfoxide (DMSO) and EtOH suspensions were added to achieve a final concentration of 1% in each solvent. Twenty-four hours after adding the metabolites, culture supernatants were removed, and 100 µL of WST-1 diluted 10-fold with fresh medium was added. After incubation at 37 °C for 30 min, absorbance was measured at 450 nm/600 nm using a multidetection mode microplate reader (Infinite 200PRO; Tecan, Männedorf, Switzerland). The cytotoxicity of the metabolites to various cells was verified by calculating the survival rate of the cells in each group relative to that of the control group, which was cultured in medium with no metabolite added and set to a value of 1.

#### Inflammatory response evaluation

epi4 was seeded into 12-well plates at 5 × 10^4^ cells/well, and pathogenic metabolites were added at various concentrations 3 days later. Thereafter, DMSO and EtOH suspensions were added to a final concentration of 1% in each solvent. The cells were harvested 6 h after addition, and total RNA was extracted using Nucleospin RNA (MACHEREY-NAGEL GmbH & Co. KG, Düren, Germany) according to the manufacturer’s protocol. cDNA synthesis was performed according to the Takara PrimeScript RT Master Mix protocol (Takara Bio Inc., Shiga, Japan). The thermal cycler used was a Takara PCR Thermal Cycler Dice Touch (TP350; Takara Bio Inc.). The RNA amount used in the reaction was 200 ng, and post-reaction samples were stored at − 30 °C. qPCR was performed using THUNDERBIRD^®^ SYBR^®^ qPCR Mix (TOYOBO, Osaka, Japan) according to the manufacturer’s instructions. The total volume of the reaction solution was 25 µL, the final primer concentration was 0.24 µM, and the cDNA amount was 2.5 µL. Primer sequences were constructed as follows, with reference to Tsutsumi et al.^45^: GAPDH-F: GCACCGTCAAGGCTGAGAAC; GAPDH-R: ATGGTGGTGAAGACGCCAGT; *Homo sapiens* interleukin 8 (IL8)-F: ACACTGCGCCAACACAGAAATTA; IL-8-R, TTTGCTTGAAGTTTCACTGGCATC. The inflammatory responsiveness of the metabolites to cells was verified by calculating the value relative to that of the control group, which was cultured in medium with no metabolite added and set to a value of 1.

### Evaluation of metabolite production from bacteria

#### Bacterial culture system

To investigate the origin of the metabolites involved in the onset and progression of periodontal disease identified, we cultured the periodontal disease-associated bacteria identified in this study as well as red and orange complex bacteria, which are already known to be closely associated with periodontal disease and examined their metabolite production. The bacteria used in this study included *P. melaninogenica* (ATCC25845), *P. intermedia* (ATCC49046), *Prevotella denticola* (ATCC33184), and *Veillonella parvula* (ATCC17745), which are constituents of the periodontal disease-associated oral microbiome identified in this study; *P. gingivalis* (W83) and (ATCC33277), the constituents of the red complex; and *F. nucleatum* subsp. *vincentii* (ATCC49256) and *F. nucleatum nucleatum* (ATCC23726) from the orange complex. Todd Hewitt Broth (BD Biosciences, Franklin Lakes, NJ, USA) containing 5 µg/mL hemin (Sigma-Aldrich), 1 µg/mL menadione (Sigma-Aldrich), and 0.5% yeast extract (BD Biosciences) was used for the liquid culture of each bacterium. To culture *V. parvula*, filter-sterilized lactic acid (FUJIFILM Wako Pure Chemical Corporation) was added at a final concentration of 2%. Each bacterium was cultured at 37℃ for 24 h under anaerobic conditions (80% nitrogen, 10% oxygen, 10% hydrogen).

#### Quantification of metabolites in bacterial culture solution

Propionate, 5-oxoproline, and homoserine were quantified using an external standard method with gas chromatography-tandem mass spectrometry (GC-MS/MS). *N*1, *N*12-diacetylspermine and citrulline were quantified using an internal standard method with multisegment injection capillary electrophoresis and triple quadrupole tandem mass spectrometry (MSI-CE-MS/MS). Succinate was quantified using an internal standard method with ion chromatography-tandem mass spectrometry (IC-MS/MS) (Supplementary Methods S1-S3).

### Statistical analysis

The oral microbiome and metabolite data were analyzed using nonparametric tests. Oral microbiome data were analyzed using the Wilcoxon rank-sum test, and significance tests were performed using the Bonferroni method. LEfSe^41^ was employed to identify bacterial species with significantly different abundance ratios and concentrations between healthy participants and those with periodontal disease. Features were considered differentially abundant if their scaled LDA analysis scores exceeded the threshold of 2.0 and had a *P*-value < 0.05. Spearman’s analysis was performed to determine the correlation between the oral microbiome and metabolites. A correlation coefficient of 0.4 or higher was determined to indicate correlation. All human gingival epithelial cell pathogenicity test data are expressed as mean ± standard deviation, and significance was assessed using Dunnett’s test, which is a parametric test. Statistical significance was considered at *P* < 0.05. All statistical analyses were performed using R (ver.4.0.2; R Foundation for Statistical Computing, Vienna, Austria).

## Electronic supplementary material

Below is the link to the electronic supplementary material.

Supplementary Material 1

## Data Availability

Microbiome analysis data (16 S rRNA gene sequences) were deposited in the DNA Data Bank of Japan (DRA015381). Subject and oral examination data and metabolome datasets collected in this study are available from the corresponding author (CI) upon reasonable request. Strengthening the Reporting of Observational Studies in Epidemiology (STROBE)^46^ was published on Zenodo DOI. The accession number is DRA015381.
