# Correction: Involvement of propionate, citrulline, homoserine, and succinate in oral microbiome metabolite-driven periodontal disease progression

**DOI:** 10.1038/s41598-026-36866-8

**Published:** 2026-01-23

**Authors:** Chikako Ishihara, Misato Sako, Kota Tsutsumi, Narumi Fujii, Daiki Hashimoto, Atsushi Sato, Yuko Ichiba, Takashi Chikazawa, Yasushi Kakizawa, Eiji Nishinaga, Akira Uchiyama

**Affiliations:** 1 Research and Development Headquarters, Lion Corporation, 7-2-1 Hirai, Edogawa-ku, Tokyo, 132-0035 Japan; 2 Section of Oral Health Promotion and Technology, Division of Oral Health, Technology and Epidemiology, Kyushu University Faculty of Dental Science, Fukuoka, Fukuoka 812-8582 Japan; 3 The Lion Foundation for Dental Health, 1-3-28 Kuramae, Taito-ku, Tokyo, 111-8644 Japan

Correction to: *Scientific Reports* 10.1038/s41598-025-91105-w, published online 28 February 2025

The original version of this Article contained an error in Figure 1, where the labels “Metabolites derived from oral microbiome” and “Metabolites with pathogenicity to human gingival cells” were swapped. The original Figure 1 and accompanying legend appear below.

The original Article has been corrected.

**Fig. 1:**
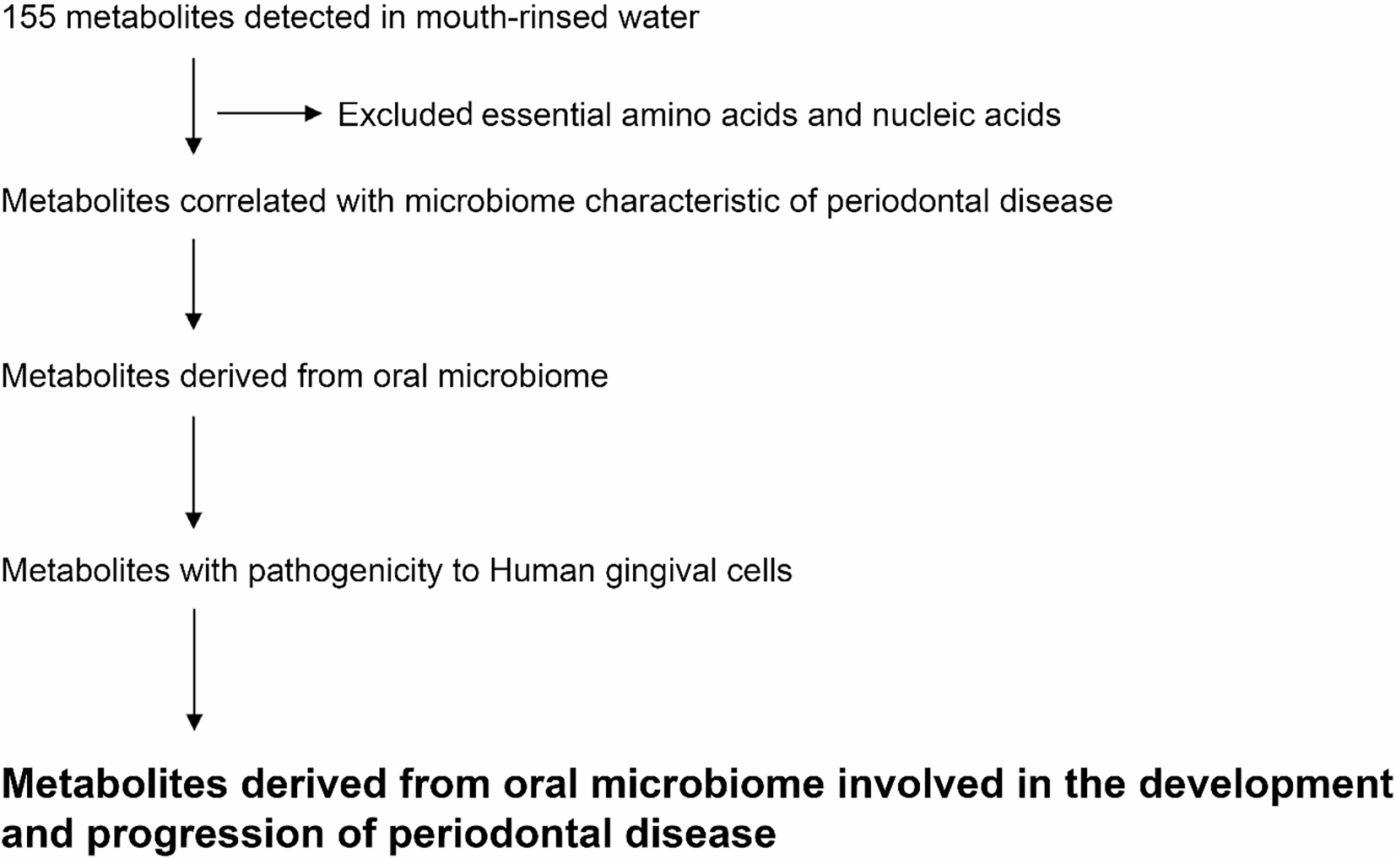
Flow diagram of metabolites involved in the development and progression of periodontal disease.

