# Supplementary material for: Involvement of propionate, citrulline, homoserine, and succinate in oral microbiome metabolite-driven periodontal disease progression
Source: Sci Rep. 2025 Feb 28;15:7149. doi: 10.1038/s41598-025-91105-w (PMC11871350; doi:10.1038/s41598-025-91105-w)
Supplement: Supplementary file 1 — Supplementary Material 1 [file 41598_2025_91105_MOESM1_ESM.docx]

**Supplementary information**

*Scientific Reports*

**Involvement of propionate, citrulline, homoserine, and succinate in oral microbiome metabolite-driven periodontal disease progression**

Chikako Ishihara^1,3,*^, Misato Sako^1^, Kota Tsutsumi^1^, Narumi Fujii^1^, Daiki Hashimoto^1,^ Atsushi Sato^1^, Yuko Ichiba^1^, Takashi Chikazawa^1^, Yasushi Kakizawa^1^, Eiji Nishinaga^1,3^, Akira Uchiyama^2,3^

^1^Research and Development Headquarters, Lion Corporation, 7-2-1 Hirai, Edogawa-ku, Tokyo 132-0035, Japan

^2^The Lion Foundation for Dental Health, 1-3-28 Kuramae, Taito-ku, Tokyo 111-8644, Japan

^3^Section of Oral Health Promotion and Technology, Division of Oral Health, Technology and Epidemiology, Kyushu University Faculty of Dental Science, Fukuoka, Fukuoka, 812-8582, Japan

^*^**Correspondence**: [c-fuji@lion.co.jp](mailto:c-fuji@lion.co.jp)





**Supplementary Figure S1**. GC-MS/MS derivatization flow. Propionate: *t-*BDMS derivatization, 5-oxoproline, homoserine: TMS derivatization.

**Supplementary Table S1**. Comparison of the periodontal disease and healthy groups.

| *species* | p_value | Health_median | Health_ave | Perio_median | Perio_ave | LDA score | Group | p value |
| --- | --- | --- | --- | --- | --- | --- | --- | --- |
| *Prevotella oris* | 0.00 | 0.01 | 0.09 | 0.15 | 0.28 | 2.90 | P | 0.00 |
| *Prevotella denticola* | 0.00 | 0.00 | 0.00 | 0.03 | 0.05 | 2.38 | P | 0.00 |
| *Tannerella forsythia* | 0.00 | 0.00 | 0.00 | 0.02 | 0.08 | 2.61 | P | 0.00 |
| *Prevotella veroralis* | 0.00 | 0.00 | 0.00 | 0.02 | 0.07 | 2.47 | P | 0.00 |
| *Parvimonas micra* | 0.00 | 0.00 | 0.02 | 0.03 | 0.09 | 2.60 | P | 0.00 |
| *Peptostreptococcaceae [XI][G-6] [Eubacterium] nodatum* | 0.00 | 0.00 | 0.00 | 0.00 | 0.03 | 2.23 | P | 0.00 |
| *Dialister invisus* | 0.00 | 0.01 | 0.02 | 0.04 | 0.05 | 2.36 | P | 0.00 |
| *Fusobacterium nucleatum subsp. vincentii* | 0.00 | 0.04 | 0.13 | 0.21 | 0.48 | 3.26 | P | 0.00 |
| *Stomatobaculum longum* | 0.00 | 0.00 | 0.01 | 0.01 | 0.05 | 2.33 | P | 0.00 |
| *Streptococcus parasanguinis clade 411* | 0.00 | 0.07 | 0.25 | 0.82 | 1.33 | 3.71 | P | 0.00 |
| *Treponema socranskii* | 0.00 | 0.00 | 0.00 | 0.02 | 0.04 | 2.29 | P | 0.00 |
| *Porphyromonas gingivalis* | 0.00 | 0.00 | 0.00 | 0.04 | 0.55 | 3.48 | P | 0.00 |
| *Fretibacterium fastidiosum* | 0.00 | 0.00 | 0.00 | 0.00 | 0.01 | 2.24 | P | 0.00 |
| *Shuttleworthia satelles* | 0.00 | 0.00 | 0.00 | 0.00 | 0.01 | 2.16 | P | 0.00 |
| *Atopobium rimae* | 0.00 | 0.00 | 0.00 | 0.01 | 0.02 | 2.18 | P | 0.00 |
| *Dialister pneumosintes* | 0.00 | 0.00 | 0.01 | 0.01 | 0.02 | 2.18 | P | 0.00 |
| *Veillonella parvula* | 0.00 | 0.22 | 0.47 | 1.19 | 1.73 | 3.73 | P | 0.00 |
| *Neisseria perflava* | 0.00 | 19.67 | 19.06 | 7.84 | 10.56 | 4.62 | H | 0.00 |
| *Atopobium parvulum* | 0.00 | 0.02 | 0.02 | 0.05 | 0.06 | 2.36 | P | 0.00 |
| *Prevotella dentalis* | 0.00 | 0.00 | 0.00 | 0.00 | 0.01 | 2.10 | P | 0.00 |
| *Pseudoramibacter alactolyticus* | 0.00 | 0.00 | 0.00 | 0.00 | 0.02 | 2.06 | P | 0.00 |
| *Neisseria meningitidis* | 0.01 | 0.00 | 0.05 | 0.00 | 0.01 | 2.36 | H | 0.01 |
| *Treponema sp. HMT 237* | 0.01 | 0.00 | 0.00 | 0.00 | 0.05 | 2.40 | P | 0.01 |
| *Filifactor alocis* | 0.01 | 0.00 | 0.01 | 0.01 | 0.15 | 2.94 | P | 0.01 |
| *Porphyromonas endodontalis* | 0.01 | 0.00 | 0.06 | 0.05 | 0.24 | 3.01 | P | 0.01 |
| *Treponema denticola* | 0.01 | 0.00 | 0.01 | 0.01 | 0.18 | 2.99 | P | 0.01 |
| *Streptococcus constellatus* | 0.01 | 0.00 | 0.01 | 0.01 | 0.07 | 2.52 | P | 0.01 |
| *Fusobacterium nucleatum subsp. nucleatum* | 0.01 | 0.00 | 0.00 | 0.00 | 0.52 | 3.46 | P | 0.01 |
| *Bacteroidetes [G-3] bacterium HMT 365* | 0.01 | 0.00 | 0.00 | 0.00 | 0.06 | 2.49 | P | 0.01 |
| *Prevotella nigrescens* | 0.01 | 0.00 | 0.00 | 0.00 | 0.01 | 2.17 | P | 0.01 |
| *Prevotella salivae* | 0.01 | 0.02 | 0.09 | 0.13 | 0.19 | 2.61 | P | 0.01 |
| *Megasphaera micronuciformis* | 0.01 | 0.02 | 0.05 | 0.06 | 0.11 | 2.40 | P | 0.02 |
| *Veillonella dispar* | 0.01 | 0.02 | 0.10 | 0.11 | 0.24 | 2.73 | P | 0.02 |
| *Mogibacterium diversum* | 0.02 | 0.00 | 0.00 | 0.00 | 0.01 | 2.47 | P | 0.01 |
| *Streptococcus sp. HMT 074* | 0.02 | 0.00 | 0.09 | 0.08 | 0.30 | 3.04 | P | 0.02 |
| *Alloprevotella sp. HMT 473* | 0.02 | 0.21 | 0.63 | 0.05 | 0.39 | 3.16 | H | 0.02 |
| *Veillonella atypica* | 0.02 | 0.08 | 0.33 | 0.40 | 0.75 | 3.20 | P | 0.02 |
| *Schaalia lingnae [Not Validly Published]* | 0.02 | 0.02 | 0.07 | 0.08 | 0.09 | 2.19 | P | 0.02 |
| *Fretibacterium sp. HMT 360* | 0.02 | 0.00 | 0.00 | 0.00 | 0.02 | 2.41 | P | 0.02 |
| *Peptostreptococcaceae [XI][G-9] [Eubacterium] brachy* | 0.02 | 0.00 | 0.02 | 0.02 | 0.07 | 2.52 | P | 0.02 |
| *Peptostreptococcaceae [XI][G-1] [Eubacterium] infirmum* | 0.02 | 0.00 | 0.00 | 0.00 | 0.01 | 2.39 | P | 0.02 |
| *Fusobacterium periodonticum* | 0.02 | 2.16 | 2.31 | 1.22 | 1.70 | 3.42 | H | 0.02 |
| *Peptostreptococcaceae [XI][G-5] [Eubacterium] saphenum* | 0.02 | 0.00 | 0.00 | 0.00 | 0.04 | 2.36 | P | 0.01 |
| *Treponema sp. HMT 262* | 0.02 | 0.00 | 0.00 | 0.00 | 0.01 | 2.14 | P | 0.01 |
| *Eggerthia catenaformis* | 0.02 | 0.00 | 0.00 | 0.00 | 0.01 | 2.07 | P | 0.01 |
| *Peptostreptococcaceae [XI][G-4] bacterium HMT 103* | 0.02 | 0.00 | 0.00 | 0.00 | 0.00 | 2.53 | P | 0.01 |
| *Fretibacterium sp. HMT 359* | 0.02 | 0.00 | 0.00 | 0.00 | 0.01 | 2.06 | P | 0.01 |
| *Solobacterium moorei* | 0.02 | 0.03 | 0.06 | 0.09 | 0.11 | 2.44 | P | 0.03 |
| *Haemophilus parainfluenzae* | 0.02 | 6.32 | 6.64 | 3.49 | 4.71 | 3.95 | H | 0.02 |
| *Prevotella histicola* | 0.03 | 0.03 | 0.19 | 0.20 | 0.66 | 3.27 | P | 0.03 |
| *Saccharibacteria (TM7) [G-1] bacterium HMT 346* | 0.03 | 0.00 | 0.01 | 0.02 | 0.03 | 2.08 | P | 0.03 |
| *Lachnospiraceae [G-2] bacterium HMT 096* | 0.03 | 0.00 | 0.02 | 0.03 | 0.04 | 2.25 | P | 0.03 |
| *Bergeyella sp. HMT 322* | 0.03 | 0.08 | 0.10 | 0.05 | 0.05 | 2.45 | H | 0.03 |
| *Streptococcus parasanguinis clade 721* | 0.03 | 0.02 | 0.10 | 0.10 | 0.32 | 3.00 | P | 0.03 |
| *Alloprevotella rava* | 0.03 | 0.00 | 0.10 | 0.03 | 0.05 | 2.60 | H | 0.03 |
| *Porphyromonas sp. HMT 278* | 0.03 | 0.00 | 0.00 | 0.00 | 0.02 | 2.30 | P | 0.03 |
| *Selenomonas sputigena* | 0.03 | 0.00 | 0.01 | 0.01 | 0.02 | 2.56 | P | 0.03 |
| *Haemophilus sp. HMT 908* | 0.03 | 0.19 | 0.37 | 0.02 | 0.27 | 2.88 | H | 0.03 |
| *Bacteroidales [G-2] bacterium HMT 274* | 0.04 | 0.00 | 0.02 | 0.02 | 0.03 |  |  | 0.04 |
| *Actinomyces sp. HMT 448* | 0.04 | 0.00 | 0.00 | 0.00 | 0.01 | 2.34 | P | 0.04 |
| *Fusobacterium sp. HMT 248* | 0.04 | 0.00 | 0.01 | 0.00 | 0.00 | 2.05 | H | 0.04 |
| *Butyrivibrio sp. HMT 455* | 0.04 | 0.00 | 0.00 | 0.00 | 0.01 | 2.36 | P | 0.04 |
| *Kingella kingae* | 0.04 | 0.00 | 0.01 | 0.00 | 0.00 | 2.48 | H | 0.03 |
| *Prevotella sp. HMT 526* | 0.05 | 0.00 | 0.00 | 0.00 | 0.01 | 2.12 | P | 0.04 |
| *Parvimonas sp. HMT 110* | 0.05 | 0.00 | 0.01 | 0.00 | 0.01 | 2.15 | H | 0.05 |
| *Streptococcus cristatus clade 578* | 0.05 | 0.04 | 0.10 | 0.22 | 0.64 | 3.42 | P | 0.05 |
| *Selenomonas sp. HMT 149* | 0.05 | 0.00 | 0.00 | 0.00 | 0.00 | 2.68 | P | 0.02 |
| *Streptococcus sobrinus* | 0.05 | 0.00 | 0.00 | 0.00 | 0.01 | 2.30 | P | 0.03 |
| *Catonella sp. HMT 451* | 0.05 | 0.00 | 0.00 | 0.00 | 0.01 | 2.10 | P | 0.03 |
| *Peptostreptococcaceae [XI][G-2] bacterium HMT 091* | 0.05 | 0.00 | 0.00 | 0.00 | 0.00 | 2.40 | P | 0.03 |
| *Clostridiales [F-1][G-1] bacterium HMT 093* | 0.05 | 0.00 | 0.00 | 0.00 | 0.02 | 2.11 | P | 0.03 |
| *Prevotella oralis* | 0.05 | 0.00 | 0.00 | 0.00 | 0.01 | 2.14 | P | 0.03 |
| *Slackia exigua* | 0.05 | 0.00 | 0.00 | 0.00 | 0.00 | 2.63 | P | 0.03 |
| *Desulfobulbus sp. HMT 041* | 0.05 | 0.00 | 0.00 | 0.00 | 0.00 | 2.47 | P | 0.03 |
| *Prevotella sp. HMT 306* | 0.05 | 0.00 | 0.05 | 0.04 | 0.18 |  |  | - |
| *Porphyromonas sp. HMT 284* | 0.05 | 0.00 | 0.05 | 0.03 | 0.16 |  |  | - |
| *Actinomyces sp. HMT 171* | 0.06 | 0.03 | 0.06 | 0.01 | 0.03 |  |  | - |
| *Streptococcus oralis subsp. tigurinus clade 070* | 0.06 | 0.00 | 0.04 | 0.02 | 0.08 |  |  | - |
| *Saccharibacteria (TM7) [G-5] bacterium HMT 356* | 0.06 | 0.00 | 0.01 | 0.02 | 0.03 |  |  | - |
| *Prevotella melaninogenica* | 0.06 | 1.00 | 1.90 | 2.41 | 3.60 |  |  | - |
| *Leptotrichia sp. HMT 221* | 0.06 | 0.00 | 0.15 | 0.07 | 0.13 |  |  | - |
| *Schaalia cardiffensis* | 0.07 | 0.00 | 0.00 | 0.00 | 0.01 |  |  | - |
| *Streptococcus oralis subsp. tigurinus clade 071* | 0.07 | 0.01 | 0.12 | 0.06 | 0.19 |  |  | - |
| *Alloprevotella sp. HMT 914* | 0.07 | 0.03 | 0.10 | 0.00 | 0.08 |  |  | - |
| *Scardovia wiggsiae* | 0.07 | 0.00 | 0.00 | 0.00 | 0.02 |  |  | - |
| *Alloprevotella sp. HMT 308* | 0.08 | 0.02 | 0.08 | 0.05 | 0.14 |  |  | - |
| *Neisseria sp. HMT 018* | 0.08 | 0.00 | 0.01 | 0.00 | 0.04 |  |  | - |
| *Treponema maltophilum* | 0.08 | 0.00 | 0.00 | 0.00 | 0.01 |  |  | - |
| *Bifidobacterium dentium* | 0.08 | 0.00 | 0.00 | 0.00 | 0.01 |  |  | - |
| *Treponema lecithinolyticum* | 0.09 | 0.00 | 0.00 | 0.00 | 0.04 |  |  | - |
| *Prevotella sp. HMT 300* | 0.09 | 0.00 | 0.01 | 0.01 | 0.02 |  |  | - |
| *Prevotella sp. HMT 309* | 0.09 | 0.00 | 0.01 | 0.00 | 0.01 |  |  | - |
| *Streptococcus intermedius* | 0.09 | 0.02 | 0.08 | 0.03 | 0.09 |  |  | - |
| *Bergeyella sp. HMT 931* | 0.09 | 0.01 | 0.04 | 0.00 | 0.02 |  |  | - |
| *Treponema sp. HMT 231* | 0.09 | 0.00 | 0.01 | 0.01 | 0.03 |  |  | - |
| *Prevotella sp. HMT 305* | 0.09 | 0.00 | 0.00 | 0.00 | 0.01 |  |  | - |
| *Schaalia odontolyticus* | 0.10 | 0.22 | 0.32 | 0.34 | 0.71 |  |  | - |
| *Streptococcus salivarius* | 0.10 | 1.13 | 2.43 | 3.55 | 4.45 |  |  | - |
| *Lachnoanaerobaculum orale* | 0.10 | 0.00 | 0.02 | 0.01 | 0.04 |  |  | - |
| *Streptococcus sp. HMT 064* | 0.10 | 0.02 | 0.21 | 0.09 | 0.22 |  |  | - |
| *Selenomonas sp. HMT 478* | 0.10 | 0.00 | 0.01 | 0.01 | 0.02 |  |  | - |
| *Enterobacter hormaechei* | 0.10 | 0.00 | 0.01 | 0.00 | 0.00 |  |  | - |
| *Lautropia mirabilis* | 0.11 | 0.95 | 1.54 | 0.43 | 0.87 |  |  | - |
| *Selenomonas noxia* | 0.11 | 0.00 | 0.00 | 0.00 | 0.01 |  |  | - |
| *Fretibacterium sp. HMT 362* | 0.11 | 0.00 | 0.00 | 0.00 | 0.01 |  |  | 0.05 |
| *Treponema sp. HMT 230* | 0.11 | 0.00 | 0.00 | 0.00 | 0.00 | 2.29 | P | 0.05 |
| *Lactococcus lactis* | 0.11 | 0.00 | 0.00 | 0.00 | 0.04 | 2.41 | P | 0.05 |
| *Bacteroidetes [G-3] bacterium HMT 280* | 0.11 | 0.00 | 0.00 | 0.00 | 0.00 | 2.07 | P | 0.05 |
| *Olsenella uli* | 0.11 | 0.00 | 0.00 | 0.00 | 0.01 | 2.39 | P | 0.05 |
| *Prevotella enoeca* | 0.11 | 0.00 | 0.00 | 0.00 | 0.00 | 2.54 | P | 0.05 |
| *Treponema sp. HMT 234* | 0.11 | 0.00 | 0.00 | 0.00 | 0.00 | 2.16 | P | 0.05 |
| *Mogibacterium timidum* | 0.11 | 0.00 | 0.00 | 0.00 | 0.00 | 2.32 | P | 0.05 |
| *Gracilibacteria (GN02) [G-1] bacterium HMT 872* | 0.12 | 0.00 | 0.01 | 0.00 | 0.00 |  |  | - |
| *Prevotella aurantiaca* | 0.12 | 0.00 | 0.06 | 0.00 | 0.03 |  |  | - |
| *Granulicatella elegans* | 0.12 | 0.11 | 0.23 | 0.02 | 0.21 |  |  | - |
| *Fusobacterium nucleatum subsp. animalis* | 0.13 | 0.05 | 0.09 | 0.09 | 0.19 |  |  | - |
| *Streptococcus sp. HMT 066* | 0.13 | 0.34 | 0.71 | 0.17 | 0.52 |  |  | - |
| *Peptostreptococcus stomatis* | 0.13 | 0.05 | 0.08 | 0.13 | 0.19 |  |  | - |
| *Streptococcus sp. HMT 057* | 0.14 | 0.02 | 0.06 | 0.04 | 0.13 |  |  | - |
| *Haemophilus sputorum* | 0.15 | 0.29 | 0.98 | 0.09 | 0.49 |  |  | - |
| *Cutibacterium acnes* | 0.15 | 0.00 | 0.00 | 0.00 | 0.00 |  |  | - |
| *Mycoplasma faucium* | 0.16 | 0.00 | 0.00 | 0.00 | 0.03 |  |  | - |
| *Peptostreptococcaceae [XI][G-7] bacterium HMT 922* | 0.16 | 0.00 | 0.02 | 0.00 | 0.00 |  |  | - |
| *Capnocytophaga sp. HMT 412* | 0.16 | 0.00 | 0.01 | 0.00 | 0.00 |  |  | - |
| *Capnocytophaga sp. HMT 864* | 0.16 | 0.00 | 0.01 | 0.00 | 0.00 |  |  | - |
| *Capnocytophaga sp. HMT 324* | 0.17 | 0.00 | 0.01 | 0.00 | 0.00 |  |  | - |
| *Haemophilus paraphrohaemolyticus* | 0.17 | 0.00 | 0.06 | 0.00 | 0.01 |  |  | - |
| *Anaeroglobus geminatus* | 0.17 | 0.00 | 0.01 | 0.00 | 0.01 |  |  | - |
| *Alloprevotella tannerae* | 0.17 | 0.03 | 0.14 | 0.08 | 0.17 |  |  | - |
| *Granulicatella adiacens* | 0.17 | 1.37 | 1.69 | 1.92 | 1.97 |  |  | - |
| *Peptostreptococcaceae [XI][G-4] bacterium HMT 369* | 0.17 | 0.00 | 0.00 | 0.00 | 0.00 |  |  | - |
| *Actinomyces dentalis* | 0.17 | 0.00 | 0.00 | 0.00 | 0.01 |  |  | - |
| *Prevotella oulorum* | 0.17 | 0.00 | 0.04 | 0.01 | 0.03 |  |  | - |
| *Prevotella shahii* | 0.18 | 0.04 | 0.11 | 0.01 | 0.06 |  |  | - |
| *Streptococcus sp. HMT 056* | 0.19 | 0.00 | 0.02 | 0.02 | 0.03 |  |  | - |
| *Selenomonas sp. HMT 136* | 0.19 | 0.00 | 0.00 | 0.00 | 0.01 |  |  | - |
| *Neisseria sp. HMT 020* | 0.20 | 0.00 | 0.00 | 0.00 | 0.01 |  |  | - |
| *Prevotella loescheii* | 0.20 | 0.00 | 0.01 | 0.00 | 0.01 |  |  | - |
| *Campylobacter gracilis* | 0.20 | 0.03 | 0.08 | 0.05 | 0.07 |  |  | - |
| *Oribacterium sp. HMT 078* | 0.20 | 0.00 | 0.00 | 0.00 | 0.01 |  |  | - |
| *Streptococcus anginosus* | 0.21 | 0.00 | 0.03 | 0.01 | 0.03 |  |  | - |
| *Bulleidia extructa* | 0.21 | 0.00 | 0.00 | 0.00 | 0.01 |  |  | - |
| *Neisseria flava* | 0.22 | 0.11 | 0.80 | 0.02 | 0.35 |  |  | - |
| *Peptococcus sp. HMT 167* | 0.22 | 0.00 | 0.00 | 0.00 | 0.00 |  |  | - |
| *Saccharibacteria (TM7) [G-4] bacterium HMT 355* | 0.22 | 0.00 | 0.00 | 0.00 | 0.00 |  |  | - |
| *Staphylococcus aureus* | 0.22 | 0.00 | 0.00 | 0.00 | 0.00 |  |  | - |
| *Leptotrichia sp. HMT 212* | 0.22 | 0.06 | 0.10 | 0.03 | 0.09 |  |  | - |
| *Neisseria macacae* | 0.23 | 0.00 | 0.79 | 0.17 | 0.85 |  |  | - |
| *Prevotella sp. HMT 313* | 0.23 | 0.01 | 0.20 | 0.10 | 0.38 |  |  | - |
| *Haemophilus pittmaniae* | 0.23 | 0.00 | 0.00 | 0.00 | 0.05 |  |  | - |
| *Peptostreptococcaceae [XI][G-7] bacterium HMT 081* | 0.23 | 0.00 | 0.00 | 0.00 | 0.00 |  |  | - |
| *Cryptobacterium curtum* | 0.23 | 0.00 | 0.00 | 0.00 | 0.00 |  |  | - |
| *Leptotrichia sp. HMT 847* | 0.23 | 0.00 | 0.00 | 0.00 | 0.01 |  |  | - |
| *Prevotella multisaccharivorax* | 0.23 | 0.00 | 0.00 | 0.00 | 0.00 |  |  | - |
| *Prevotella sp. HMT 304* | 0.23 | 0.00 | 0.00 | 0.00 | 0.13 |  |  | - |
| *Aggregatibacter sp. HMT 513* | 0.23 | 0.00 | 0.00 | 0.00 | 0.01 |  |  | - |
| *Capnocytophaga sp. HMT 323* | 0.23 | 0.00 | 0.00 | 0.00 | 0.00 |  |  | - |
| *Schaalia sp. HMT 178* | 0.23 | 0.00 | 0.00 | 0.00 | 0.00 |  |  | - |
| *Bacteroidetes [G-6] bacterium HMT 516* | 0.23 | 0.00 | 0.00 | 0.00 | 0.01 |  |  | - |
| *Stomatobaculum sp. HMT 910* | 0.23 | 0.00 | 0.00 | 0.00 | 0.00 |  |  | - |
| *Peptoniphilaceae [G-1] bacterium HMT 113* | 0.23 | 0.00 | 0.00 | 0.00 | 0.00 |  |  | - |
| *Fusobacterium sp. HMT 204* | 0.24 | 0.00 | 0.01 | 0.00 | 0.01 |  |  | - |
| *Mycoplasma salivarium* | 0.24 | 0.00 | 0.00 | 0.00 | 0.00 |  |  | - |
| *Fusobacterium sp. HMT 370* | 0.25 | 0.00 | 0.00 | 0.00 | 0.01 |  |  | - |
| *Actinomyces massiliensis* | 0.27 | 0.01 | 0.02 | 0.01 | 0.01 |  |  | - |
| *Capnocytophaga leadbetteri* | 0.27 | 0.04 | 0.08 | 0.03 | 0.05 |  |  | - |
| *Saccharibacteria (TM7) [G-1] bacterium HMT 347* | 0.27 | 0.00 | 0.01 | 0.00 | 0.01 |  |  | - |
| *Schaalia georgiae* | 0.28 | 0.00 | 0.00 | 0.00 | 0.00 |  |  | - |
| *Prevotella maculosa* | 0.28 | 0.00 | 0.02 | 0.01 | 0.02 |  |  | - |
| *Capnocytophaga sp. HMT 863* | 0.28 | 0.00 | 0.00 | 0.00 | 0.00 |  |  | - |
| *Bacteroidaceae [G-1] bacterium HMT 272* | 0.28 | 0.00 | 0.00 | 0.00 | 0.00 |  |  | - |
| *Campylobacter sp. HMT 044* | 0.28 | 0.00 | 0.00 | 0.00 | 0.01 |  |  | - |
| *Kingella sp. HMT 012* | 0.28 | 0.00 | 0.01 | 0.00 | 0.00 |  |  | - |
| *Cardiobacterium valvarum* | 0.29 | 0.01 | 0.03 | 0.00 | 0.02 |  |  | - |
| *Prevotella intermedia* | 0.29 | 0.00 | 0.03 | 0.00 | 0.16 |  |  | - |
| *Leptotrichia sp. HMT 498* | 0.29 | 0.00 | 0.03 | 0.00 | 0.09 |  |  | - |
| *Absconditabacteria (SR1) [G-1] bacterium HMT 875* | 0.29 | 0.03 | 0.26 | 0.01 | 0.08 |  |  | - |
| *Capnocytophaga sp. HMT 336* | 0.30 | 0.00 | 0.02 | 0.00 | 0.01 |  |  | - |
| *Capnocytophaga sp. HMT 335* | 0.30 | 0.00 | 0.02 | 0.01 | 0.03 |  |  | - |
| *Lachnospiraceae [G-3] bacterium HMT 100* | 0.30 | 0.01 | 0.03 | 0.01 | 0.02 |  |  | - |
| *Gemella haemolysans* | 0.31 | 0.29 | 0.58 | 0.57 | 1.06 |  |  | - |
| *Fusobacterium hwasookii* | 0.31 | 0.02 | 0.08 | 0.01 | 0.01 |  |  | - |
| *Mitsuokella sp. HMT 131* | 0.31 | 0.00 | 0.00 | 0.00 | 0.00 |  |  | - |
| *Porphyromonas pasteri* | 0.32 | 1.48 | 1.84 | 1.04 | 1.47 |  |  | - |
| *Corynebacterium durum* | 0.32 | 0.05 | 0.08 | 0.04 | 0.08 |  |  | - |
| *Actinomyces oris* | 0.32 | 0.00 | 0.02 | 0.00 | 0.01 |  |  | - |
| *Prevotella sp. HMT 315* | 0.33 | 0.00 | 0.00 | 0.00 | 0.00 |  |  | - |
| *Haemophilus parahaemolyticus* | 0.34 | 0.04 | 0.30 | 0.01 | 0.07 |  |  | - |
| *Leptotrichia sp. HMT 215* | 0.34 | 0.09 | 0.18 | 0.11 | 0.14 |  |  | - |
| *Prevotella baroniae* | 0.34 | 0.00 | 0.02 | 0.00 | 0.01 |  |  | - |
| *Capnocytophaga granulosa* | 0.35 | 0.03 | 0.11 | 0.02 | 0.05 |  |  | - |
| *Leptotrichia sp. HMT 218* | 0.35 | 0.00 | 0.01 | 0.00 | 0.01 |  |  | - |
| *Lactobacillus gasseri* | 0.35 | 0.00 | 0.02 | 0.00 | 0.00 |  |  | - |
| *Haemophilus sp. HMT 036* | 0.35 | 0.05 | 0.26 | 0.01 | 0.10 |  |  | - |
| *Corynebacterium matruchotii* | 0.35 | 0.00 | 0.07 | 0.01 | 0.05 |  |  | - |
| *Leptotrichia hofstadii* | 0.36 | 0.01 | 0.06 | 0.02 | 0.08 |  |  | - |
| *Lachnoanaerobaculum saburreum* | 0.36 | 0.00 | 0.02 | 0.01 | 0.02 |  |  | - |
| *Haemophilus haemolyticus* | 0.36 | 0.03 | 0.21 | 0.01 | 0.22 |  |  | - |
| *Schaalia sp. HMT 180* | 0.36 | 0.02 | 0.06 | 0.05 | 0.12 |  |  | - |
| *Alloprevotella sp. HMT 913* | 0.37 | 0.00 | 0.01 | 0.00 | 0.00 |  |  | - |
| *Streptococcus australis* | 0.37 | 0.41 | 0.75 | 0.31 | 0.62 |  |  | - |
| *Streptococcus sanguinis* | 0.37 | 0.67 | 1.01 | 0.55 | 0.65 |  |  | - |
| *Streptococcus mutans* | 0.38 | 0.00 | 0.04 | 0.00 | 0.03 |  |  | - |
| *Selenomonas artemidis* | 0.38 | 0.00 | 0.00 | 0.00 | 0.01 |  |  | - |
| *Prevotella sp. HMT 475* | 0.39 | 0.00 | 0.01 | 0.00 | 0.00 |  |  | - |
| *Prevotella sp. HMT 314* | 0.39 | 0.00 | 0.01 | 0.00 | 0.02 |  |  | - |
| *Saccharibacteria (TM7) [G-1] bacterium HMT 352* | 0.40 | 0.19 | 0.22 | 0.12 | 0.24 |  |  | - |
| *Catonella morbi* | 0.40 | 0.08 | 0.08 | 0.05 | 0.08 |  |  | - |
| *Saccharibacteria (TM7) [G-1] bacterium HMT 348* | 0.40 | 0.00 | 0.03 | 0.01 | 0.03 |  |  | - |
| *Gemella morbillorum* | 0.40 | 0.23 | 0.52 | 0.41 | 0.62 |  |  | - |
| *Prevotella sp. HMT 942* | 0.40 | 0.00 | 0.00 | 0.00 | 0.01 |  |  | - |
| *Oribacterium sinus* | 0.41 | 0.12 | 0.18 | 0.12 | 0.14 |  |  | - |
| *Bacteroidetes [G-5] bacterium HMT 511* | 0.41 | 0.00 | 0.01 | 0.00 | 0.01 |  |  | - |
| *Streptococcus pneumoniae* | 0.41 | 0.01 | 0.01 | 0.00 | 0.01 |  |  | - |
| *Rothia aeria* | 0.42 | 0.78 | 1.25 | 0.68 | 1.19 |  |  | - |
| *Saccharibacteria (TM7) [G-1] bacterium HMT 957* | 0.42 | 0.00 | 0.01 | 0.00 | 0.02 |  |  | - |
| *Bergeyella sp. HMT 206* | 0.42 | 0.00 | 0.14 | 0.00 | 0.06 |  |  | - |
| *Saccharibacteria (TM7) [G-1] bacterium HMT 349* | 0.42 | 0.00 | 0.02 | 0.00 | 0.02 |  |  | - |
| *Streptococcus oralis subsp. dentisani clade 058* | 0.43 | 0.87 | 1.07 | 0.60 | 1.03 |  |  | - |
| *Actinomyces graevenitzii* | 0.43 | 0.15 | 0.48 | 0.30 | 0.56 |  |  | - |
| *Streptococcus gordonii* | 0.44 | 0.02 | 0.08 | 0.03 | 0.16 |  |  | - |
| *Porphyromonas catoniae* | 0.44 | 0.01 | 0.03 | 0.00 | 0.02 |  |  | - |
| *Cardiobacterium hominis* | 0.44 | 0.03 | 0.05 | 0.02 | 0.06 |  |  | - |
| *Fusobacterium sp. HMT 203* | 0.44 | 0.00 | 0.08 | 0.01 | 0.04 |  |  | - |
| *Prevotella pallens* | 0.45 | 0.28 | 0.43 | 0.34 | 0.53 |  |  | - |
| *Campylobacter showae* | 0.46 | 0.04 | 0.08 | 0.02 | 0.05 |  |  | - |
| *Veillonella sp. HMT 780* | 0.46 | 0.01 | 0.12 | 0.00 | 0.08 |  |  | - |
| *Prevotella sp. HMT 472* | 0.46 | 0.01 | 0.02 | 0.00 | 0.04 |  |  | - |
| *Veillonella rogosae* | 0.47 | 1.40 | 1.52 | 1.09 | 1.48 |  |  | - |
| *Prevotella sp. HMT 293* | 0.48 | 0.00 | 0.00 | 0.00 | 0.00 |  |  | - |
| *Sneathia amnii [Not Validly Published]* | 0.48 | 0.00 | 0.01 | 0.00 | 0.00 |  |  | - |
| *Klebsiella aerogenes* | 0.48 | 0.00 | 0.00 | 0.00 | 0.00 |  |  | - |
| *Prevotella sp. HMT 292* | 0.48 | 0.00 | 0.00 | 0.00 | 0.00 |  |  | - |
| *Actinomyces israelii* | 0.48 | 0.00 | 0.00 | 0.00 | 0.00 |  |  | - |
| *Streptococcus sinensis* | 0.48 | 0.00 | 0.01 | 0.00 | 0.00 |  |  | - |
| *Bifidobacterium animalis* | 0.48 | 0.00 | 0.00 | 0.00 | 0.00 |  |  | - |
| *Actinomyces timonensis* | 0.48 | 0.00 | 0.00 | 0.00 | 0.00 |  |  | - |
| *Capnocytophaga haemolytica* | 0.48 | 0.00 | 0.00 | 0.00 | 0.00 |  |  | - |
| *Bacillus anthracis* | 0.48 | 0.00 | 0.00 | 0.00 | 0.00 |  |  | - |
| *Enterococcus faecalis* | 0.48 | 0.00 | 0.00 | 0.00 | 0.00 |  |  | - |
| *Cutibacterium granulosum* | 0.48 | 0.00 | 0.00 | 0.00 | 0.00 |  |  | - |
| *Veillonella denticariosi* | 0.48 | 0.00 | 0.00 | 0.00 | 0.00 |  |  | - |
| *Capnocytophaga sp. HMT 902* | 0.48 | 0.00 | 0.00 | 0.00 | 0.00 |  |  | - |
| *Acidovorax temperans* | 0.48 | 0.00 | 0.00 | 0.00 | 0.00 |  |  | - |
| *Lawsonella clevelandensis* | 0.48 | 0.00 | 0.00 | 0.00 | 0.00 |  |  | - |
| *Peptidiphaga sp. HMT 183* | 0.49 | 0.01 | 0.03 | 0.01 | 0.02 |  |  | - |
| *Leptotrichia sp. HMT 417* | 0.49 | 0.06 | 0.22 | 0.08 | 0.22 |  |  | - |
| *Streptococcus oralis subsp. oralis* | 0.49 | 0.03 | 0.16 | 0.10 | 0.26 |  |  | - |
| *Bacteroides heparinolyticus* | 0.49 | 0.00 | 0.00 | 0.00 | 0.01 |  |  | - |
| *Erysipelotrichaceae [G-1] bacterium HMT 905* | 0.49 | 0.00 | 0.00 | 0.00 | 0.00 |  |  | - |
| *Treponema amylovorum* | 0.49 | 0.00 | 0.00 | 0.00 | 0.07 |  |  | - |
| *Treponema sp. HMT 257* | 0.49 | 0.00 | 0.00 | 0.00 | 0.00 |  |  | - |
| *Prevotella marshii* | 0.49 | 0.00 | 0.00 | 0.00 | 0.00 |  |  | - |
| *Parascardovia denticolens* | 0.49 | 0.00 | 0.00 | 0.00 | 0.00 |  |  | - |
| *Desulfovibrio sp. HMT 040* | 0.49 | 0.00 | 0.00 | 0.00 | 0.00 |  |  | - |
| *Porphyromonas uenonis* | 0.49 | 0.00 | 0.00 | 0.00 | 0.00 |  |  | - |
| *Fretibacterium sp. HMT 361* | 0.49 | 0.00 | 0.00 | 0.00 | 0.00 |  |  | - |
| *Treponema parvum* | 0.49 | 0.00 | 0.00 | 0.00 | 0.00 |  |  | - |
| *Treponema sp. HMT 253* | 0.49 | 0.00 | 0.00 | 0.00 | 0.00 |  |  | - |
| *Erysipelotrichaceae [G-1] bacterium HMT 904* | 0.49 | 0.00 | 0.00 | 0.00 | 0.00 |  |  | - |
| *Anaerolineae [G-1] bacterium HMT 439* | 0.49 | 0.00 | 0.00 | 0.00 | 0.00 |  |  | - |
| *Selenomonas sp. HMT 134* | 0.50 | 0.00 | 0.00 | 0.00 | 0.00 |  |  | - |
| *Leptotrichia goodfellowii* | 0.51 | 0.00 | 0.01 | 0.00 | 0.01 |  |  | - |
| *Streptococcus vestibularis* | 0.51 | 0.00 | 0.02 | 0.00 | 0.10 |  |  | - |
| *Rothia dentocariosa* | 0.52 | 0.63 | 1.17 | 0.48 | 1.21 |  |  | - |
| *Streptococcus infantis clade 431* | 0.52 | 0.88 | 1.12 | 0.83 | 0.95 |  |  | - |
| *Absconditabacteria (SR1) [G-1] bacterium HMT 345* | 0.52 | 0.00 | 0.04 | 0.00 | 0.02 |  |  | - |
| *Capnocytophaga gingivalis* | 0.52 | 0.06 | 0.09 | 0.04 | 0.10 |  |  | - |
| *Arachnia propionica* | 0.52 | 0.03 | 0.04 | 0.02 | 0.04 |  |  | - |
| *Prevotella pleuritidis* | 0.53 | 0.00 | 0.01 | 0.00 | 0.01 |  |  | - |
| *Schaalia sp. HMT 172* | 0.53 | 0.03 | 0.41 | 0.06 | 0.73 |  |  | - |
| *Butyrivibrio sp. HMT 080* | 0.54 | 0.00 | 0.00 | 0.00 | 0.00 |  |  | - |
| *Saccharibacteria (TM7) [G-1] bacterium HMT 869* | 0.54 | 0.00 | 0.01 | 0.00 | 0.01 |  |  | - |
| *Prevotella micans* | 0.55 | 0.00 | 0.01 | 0.00 | 0.01 |  |  | - |
| *Peptostreptococcaceae [XI][G-1] [Eubacterium] sulci* | 0.55 | 0.03 | 0.06 | 0.04 | 0.10 |  |  | - |
| *Leptotrichia sp. HMT 223* | 0.55 | 0.00 | 0.01 | 0.00 | 0.05 |  |  | - |
| *Ottowia sp. HMT 894* | 0.57 | 0.01 | 0.03 | 0.00 | 0.02 |  |  | - |
| *Aggregatibacter sp. HMT 458* | 0.57 | 0.04 | 0.08 | 0.02 | 0.11 |  |  | - |
| *Gemella sanguinis* | 0.57 | 1.13 | 1.21 | 0.81 | 1.26 |  |  | - |
| *Capnocytophaga sp. HMT 332* | 0.57 | 0.00 | 0.00 | 0.00 | 0.01 |  |  | - |
| *Capnocytophaga sp. HMT 326* | 0.57 | 0.00 | 0.01 | 0.00 | 0.01 |  |  | - |
| *Streptococcus oralis subsp. dentisani clade 398* | 0.57 | 0.10 | 0.87 | 0.08 | 0.58 |  |  | - |
| *Actinomyces sp. HMT 170* | 0.58 | 0.01 | 0.01 | 0.01 | 0.02 |  |  | - |
| *Streptococcus sp. HMT 061* | 0.58 | 0.07 | 0.16 | 0.05 | 0.35 |  |  | - |
| *Oribacterium parvum* | 0.59 | 0.00 | 0.01 | 0.00 | 0.01 |  |  | - |
| *Neisseria bacilliformis* | 0.60 | 0.00 | 0.01 | 0.00 | 0.01 |  |  | - |
| *Leptotrichia wadei* | 0.60 | 0.00 | 0.06 | 0.02 | 0.05 |  |  | - |
| *Mycoplasma orale* | 0.61 | 0.00 | 0.00 | 0.00 | 0.00 |  |  | - |
| *Aggregatibacter paraphrophilus* | 0.61 | 0.00 | 0.02 | 0.00 | 0.01 |  |  | - |
| *Actinomyces johnsonii* | 0.61 | 0.00 | 0.01 | 0.00 | 0.01 |  |  | - |
| *Peptidiphaga gingivicola* | 0.61 | 0.00 | 0.00 | 0.00 | 0.00 |  |  | - |
| *Veillonella sp. HMT 917* | 0.61 | 0.00 | 0.01 | 0.00 | 0.01 |  |  | - |
| *Saccharibacteria (TM7) [G-2] bacterium HMT 350* | 0.61 | 0.00 | 0.01 | 0.00 | 0.00 |  |  | - |
| *Actinomyces sp. HMT 414* | 0.61 | 0.00 | 0.00 | 0.00 | 0.00 |  |  | - |
| *Leptotrichia buccalis* | 0.61 | 0.02 | 0.04 | 0.01 | 0.04 |  |  | - |
| *Fusobacterium nucleatum subsp. polymorphum* | 0.61 | 0.12 | 0.15 | 0.07 | 0.16 |  |  | - |
| *Aggregatibacter segnis* | 0.61 | 0.00 | 0.01 | 0.00 | 0.02 |  |  | - |
| *Prevotella nanceiensis* | 0.61 | 0.23 | 0.47 | 0.34 | 0.40 |  |  | - |
| *Porphyromonas sp. HMT 275* | 0.62 | 0.01 | 0.16 | 0.00 | 0.07 |  |  | - |
| *Actinomyces sp. HMT 175* | 0.62 | 0.00 | 0.02 | 0.00 | 0.01 |  |  | - |
| *Campylobacter rectus* | 0.63 | 0.00 | 0.00 | 0.00 | 0.01 |  |  | - |
| *Kingella denitrificans* | 0.64 | 0.00 | 0.05 | 0.01 | 0.04 |  |  | - |
| *Abiotrophia defectiva* | 0.64 | 0.00 | 0.08 | 0.01 | 0.07 |  |  | - |
| *Leptotrichia sp. HMT 225* | 0.64 | 0.01 | 0.03 | 0.01 | 0.02 |  |  | - |
| *Absconditabacteria (SR1) [G-1] bacterium HMT 874* | 0.64 | 0.00 | 0.01 | 0.00 | 0.01 |  |  | - |
| *Johnsonella ignava* | 0.65 | 0.00 | 0.00 | 0.00 | 0.00 |  |  | - |
| *Prevotella sp. HMT 317* | 0.65 | 0.01 | 0.03 | 0.01 | 0.04 |  |  | - |
| *Aggregatibacter sp. HMT 512* | 0.66 | 0.00 | 0.01 | 0.00 | 0.04 |  |  | - |
| *Saccharibacteria (TM7) [G-6] bacterium HMT 870* | 0.66 | 0.05 | 0.07 | 0.06 | 0.10 |  |  | - |
| *Tannerella sp. HMT 808* | 0.68 | 0.00 | 0.00 | 0.00 | 0.00 |  |  | - |
| *Neisseria elongata* | 0.68 | 0.21 | 0.36 | 0.21 | 0.39 |  |  | - |
| *Oribacterium asaccharolyticum* | 0.69 | 0.04 | 0.05 | 0.03 | 0.05 |  |  | - |
| *Porphyromonas sp. HMT 285* | 0.69 | 0.00 | 0.00 | 0.00 | 0.01 |  |  | - |
| *Rothia mucilaginosa* | 0.70 | 2.52 | 4.80 | 3.14 | 3.80 |  |  | - |
| *Aggregatibacter sp. HMT 898* | 0.72 | 0.00 | 0.01 | 0.00 | 0.01 |  |  | - |
| *Schaalia sp. HMT 877* | 0.73 | 0.00 | 0.01 | 0.00 | 0.01 |  |  | - |
| *Prevotella sp. HMT 515* | 0.73 | 0.00 | 0.00 | 0.00 | 0.00 |  |  | - |
| *Leptotrichia shahii* | 0.73 | 0.00 | 0.00 | 0.00 | 0.01 |  |  | - |
| *Selenomonas sp. HMT 138* | 0.73 | 0.00 | 0.00 | 0.00 | 0.00 |  |  | - |
| *Kingella sp. HMT 932* | 0.74 | 0.00 | 0.02 | 0.00 | 0.01 |  |  | - |
| *Treponema medium* | 0.74 | 0.00 | 0.01 | 0.00 | 0.00 |  |  | - |
| *Bacillus subtilis* | 0.74 | 0.00 | 0.01 | 0.00 | 0.00 |  |  | - |
| *Escherichia coli* | 0.74 | 0.00 | 0.01 | 0.00 | 0.00 |  |  | - |
| *Actinomyces sp. HMT 896* | 0.74 | 0.00 | 0.00 | 0.00 | 0.00 |  |  | - |
| *Neisseriaceae [G-1] bacterium HMT 174* | 0.74 | 0.00 | 0.00 | 0.00 | 0.00 |  |  | - |
| *Others(<99%)* | 0.76 | 18.05 | 17.99 | 18.35 | 19.44 |  |  | - |
| *Capnocytophaga sp. HMT 901* | 0.76 | 0.00 | 0.00 | 0.00 | 0.00 |  |  | - |
| *Fusobacterium naviforme* | 0.76 | 0.00 | 0.00 | 0.00 | 0.02 |  |  | - |
| *Streptococcus mitis* | 0.77 | 5.18 | 7.23 | 4.92 | 7.30 |  |  | - |
| *Eikenella corrodens* | 0.77 | 0.03 | 0.05 | 0.04 | 0.06 |  |  | - |
| *Prevotella buccae* | 0.78 | 0.00 | 0.00 | 0.00 | 0.00 |  |  | - |
| *Prevotella scopos* | 0.80 | 0.00 | 0.01 | 0.00 | 0.07 |  |  | - |
| *Ruminococcaceae [G-2] bacterium HMT 085* | 0.80 | 0.03 | 0.04 | 0.03 | 0.05 |  |  | - |
| *Kingella oralis* | 0.80 | 0.01 | 0.05 | 0.01 | 0.06 |  |  | - |
| *Selenomonas flueggei* | 0.80 | 0.00 | 0.01 | 0.00 | 0.00 |  |  | - |
| *Streptococcus thermophilus* | 0.82 | 0.00 | 0.06 | 0.00 | 0.01 |  |  | - |
| *Alloprevotella sp. HMT 912* | 0.83 | 0.00 | 0.00 | 0.00 | 0.00 |  |  | - |
| *Treponema vincentii* | 0.83 | 0.00 | 0.00 | 0.00 | 0.00 |  |  | - |
| *Actinomyces sp. HMT 169* | 0.86 | 0.05 | 0.12 | 0.04 | 0.51 |  |  | - |
| *Streptococcus infantis clade 638* | 0.86 | 0.69 | 1.19 | 0.80 | 0.97 |  |  | - |
| *Actinomyces naeslundii* | 0.86 | 0.04 | 0.08 | 0.03 | 0.05 |  |  | - |
| *Tannerella sp. HMT 286* | 0.87 | 0.01 | 0.03 | 0.01 | 0.03 |  |  | - |
| *Streptococcus cristatus clade 886* | 0.87 | 0.00 | 0.00 | 0.00 | 0.01 |  |  | - |
| *Lachnospiraceae [G-8] bacterium HMT 500* | 0.87 | 0.00 | 0.00 | 0.00 | 0.01 |  |  | - |
| *Mitsuokella sp. HMT 521* | 0.87 | 0.00 | 0.00 | 0.00 | 0.00 |  |  | - |
| *Schaalia meyeri* | 0.89 | 0.00 | 0.00 | 0.00 | 0.01 |  |  | - |
| *Campylobacter concisus* | 0.89 | 0.13 | 0.16 | 0.15 | 0.18 |  |  | - |
| *Lachnoanaerobaculum umeaense* | 0.89 | 0.01 | 0.02 | 0.02 | 0.02 |  |  | - |
| *Arachnia rubra* | 0.90 | 0.03 | 0.04 | 0.02 | 0.04 |  |  | - |
| *Neisseria sicca* | 0.90 | 0.00 | 0.09 | 0.00 | 0.02 |  |  | - |
| *Streptococcus peroris* | 0.90 | 0.00 | 0.00 | 0.00 | 0.00 |  |  | - |
| *Stomatobaculum sp. HMT 097* | 0.91 | 0.06 | 0.08 | 0.05 | 0.10 |  |  | - |
| *Porphyromonas sp. HMT 930* | 0.91 | 0.00 | 0.00 | 0.00 | 0.02 |  |  | - |
| *Leptotrichia hongkongensis* | 0.92 | 0.04 | 0.15 | 0.04 | 0.12 |  |  | - |
| *Selenomonas dianae* | 0.92 | 0.00 | 0.00 | 0.00 | 0.00 |  |  | - |
| *Prevotella sp. HMT 301* | 0.93 | 0.00 | 0.00 | 0.00 | 0.00 |  |  | - |
| *Leptotrichia sp. HMT 219* | 0.94 | 0.00 | 0.02 | 0.00 | 0.01 |  |  | - |
| *Ruminococcaceae [G-1] bacterium HMT 075* | 0.94 | 0.02 | 0.03 | 0.02 | 0.05 |  |  | - |
| *Leptotrichia sp. HMT 392* | 0.95 | 0.01 | 0.07 | 0.01 | 0.03 |  |  | - |
| *Peptostreptococcaceae [XI][G-7] [Eubacterium] yurii subsps. yurii & margaretiae* | 0.95 | 0.00 | 0.01 | 0.00 | 0.00 |  |  | - |
| *Veillonellaceae [G-1] bacterium HMT 155* | 0.97 | 0.00 | 0.00 | 0.00 | 0.00 |  |  | - |
| *Capnocytophaga sp. HMT 338* | 0.97 | 0.00 | 0.00 | 0.00 | 0.00 |  |  | - |
| *Capnocytophaga sp. HMT 380* | 0.98 | 0.00 | 0.02 | 0.00 | 0.01 |  |  | - |
| *Saccharibacteria (TM7) [G-3] bacterium HMT 351* | 0.98 | 0.01 | 0.02 | 0.01 | 0.03 |  |  | - |
| *Neisseria oralis* | 0.98 | 0.13 | 0.63 | 0.08 | 0.68 |  |  | - |
| *Capnocytophaga sputigena* | 0.98 | 0.08 | 0.23 | 0.09 | 0.17 |  |  | - |
| *Streptococcus sp. HMT 423* | 0.98 | 0.77 | 1.15 | 0.75 | 1.11 |  |  | - |
| *Aggregatibacter aphrophilus* | 1.00 | 0.00 | 0.00 | 0.00 | 0.02 |  |  | - |
| *Leptotrichia sp. HMT 463* | 1.00 | 0.00 | 0.00 | 0.00 | 0.01 |  |  | - |
| *Aggregatibacter sp. HMT 949* | 1.00 | 0.00 | 0.00 | 0.00 | 0.00 |  |  | - |
| *Johnsonella sp. HMT 166* | 1.00 | 0.00 | 0.00 | 0.00 | 0.00 |  |  | - |
| *Lactobacillus salivarius* | 1.00 | 0.00 | 0.01 | 0.00 | 0.00 |  |  | - |
| *Stenotrophomonas maltophilia* | 1.00 | 0.00 | 0.00 | 0.00 | 0.00 |  |  | - |
| *Bifidobacterium longum* | 1.00 | 0.00 | 0.00 | 0.00 | 0.00 |  |  | - |
| *Propionibacterium acidifaciens* | 1.00 | 0.00 | 0.00 | 0.00 | 0.00 |  |  | - |
| *Selenomonas sp. HMT 892* | 1.00 | 0.00 | 0.00 | 0.00 | 0.00 |  |  | - |
| *Lactobacillus ultunensis* | 1.00 | 0.00 | 0.00 | 0.00 | 0.00 |  |  | - |
| *Atopobium sp. HMT 416* | 1.00 | 0.00 | 0.00 | 0.00 | 0.00 |  |  | - |
| *Catonella sp. HMT 164* | 1.00 | 0.00 | 0.00 | 0.00 | 0.00 |  |  | - |
| *Oribacterium sp. HMT 102* | 1.00 | 0.00 | 0.00 | 0.00 | 0.00 |  |  | - |
| *Treponema sp. HMT 236* | 1.00 | 0.00 | 0.00 | 0.00 | 0.00 |  |  | - |
| *Treponema sp. HMT 268* | 1.00 | 0.00 | 0.00 | 0.00 | 0.00 |  |  | - |
| *Prevotella saccharolytica* | 1.00 | 0.00 | 0.01 | 0.00 | 0.01 |  |  | - |
| *Saccharibacteria (TM7) [G-8] bacterium HMT 955* | 1.00 | 0.00 | 0.00 | 0.00 | 0.00 |  |  | - |
| *Finegoldia magna* | 1.00 | 0.00 | 0.00 | 0.00 | 0.00 |  |  | - |
| *Actinomyces gerencseriae* | 1.00 | 0.00 | 0.01 | 0.00 | 0.00 |  |  | - |
| *Selenomonas sp. HMT 137* | 1.00 | 0.00 | 0.00 | 0.00 | 0.00 |  |  | - |
| *Bergeyella sp. HMT 900* | 1.00 | 0.00 | 0.00 | 0.00 | 0.00 |  |  | - |
| *Prevotella bivia* | 1.00 | 0.00 | 0.00 | 0.00 | 0.00 |  |  | - |
| *Lachnospiraceae [G-2] bacterium HMT 088* | 1.00 | 0.00 | 0.00 | 0.00 | 0.00 |  |  | - |
| *Veillonellaceae [G-1] bacterium HMT 150* | 1.00 | 0.00 | 0.00 | 0.00 | 0.00 |  |  | - |
| *Haemophilus influenzae* | 1.00 | 0.00 | 0.00 | 0.00 | 0.00 |  |  | - |
| *Veillonellaceae [G-1] bacterium HMT 145* | 1.00 | 0.00 | 0.00 | 0.00 | 0.00 |  |  | - |
| *Selenomonas sp. HMT 126* | 1.00 | 0.00 | 0.00 | 0.00 | 0.00 |  |  | - |
| *Stomatobaculum sp. HMT 373* | 1.00 | 0.00 | 0.00 | 0.00 | 0.00 |  |  | - |
| *Selenomonas sp. HMT 481* | 1.00 | 0.00 | 0.00 | 0.00 | 0.00 |  |  | - |
| *Treponema sp. HMT 256* | 1.00 | 0.00 | 0.00 | 0.00 | 0.00 |  |  | - |
| *Selenomonas sp. HMT 919* | 1.00 | 0.00 | 0.00 | 0.00 | 0.00 |  |  | - |
| *Actinomyces sp. HMT 525* | 1.00 | 0.00 | 0.00 | 0.00 | 0.00 |  |  | - |
| *Campylobacter curvus* | 1.00 | 0.00 | 0.00 | 0.00 | 0.00 |  |  | - |
| *Selenomonas sp. HMT 937* | 1.00 | 0.00 | 0.00 | 0.00 | 0.00 |  |  | - |
| *Peptoniphilus lacrimalis* | 1.00 | 0.00 | 0.00 | 0.00 | 0.00 |  |  | - |
| *Olsenella sp. HMT 807* | 1.00 | 0.00 | 0.00 | 0.00 | 0.00 |  |  | - |
| *Fusobacterium necrophorum* | 1.00 | 0.00 | 0.00 | 0.00 | 0.00 |  |  | - |
| *Actinomyces sp. HMT 897* | 1.00 | 0.00 | 0.00 | 0.00 | 0.00 |  |  | - |
| *Veillonellaceae [G-1] bacterium HMT 135* | 1.00 | 0.00 | 0.00 | 0.00 | 0.00 |  |  | - |
| *Desulfomicrobium orale* | 1.00 | 0.00 | 0.00 | 0.00 | 0.00 |  |  | - |
| *Enterobacter cancerogenus* | 1.00 | 0.00 | 0.00 | 0.00 | 0.00 |  |  | - |
| *Olsenella profusa* | 1.00 | 0.00 | 0.00 | 0.00 | 0.00 |  |  | - |
| *Treponema sp. HMT 260* | 1.00 | 0.00 | 0.00 | 0.00 | 0.00 |  |  | - |
| *Kingella sp. HMT 459* | 1.00 | 0.00 | 0.00 | 0.00 | 0.00 |  |  | - |
| *Staphylococcus epidermidis* | 1.00 | 0.00 | 0.00 | 0.00 | 0.00 |  |  | - |
| *Aggregatibacter actinomycetemcomitans* | 1.00 | 0.00 | 0.00 | 0.00 | 0.00 |  |  | - |
| *Delftia acidovorans* | 1.00 | 0.00 | 0.00 | 0.00 | 0.00 |  |  | - |
| *Lachnospiraceae [G-7] bacterium HMT 086* | 1.00 | 0.00 | 0.00 | 0.00 | 0.00 |  |  | - |
| *Kluyvera ascorbata* | 1.00 | 0.00 | 0.00 | 0.00 | 0.00 |  |  | - |
| *Burkholderia cepacia* | 1.00 | 0.00 | 0.00 | 0.00 | 0.00 |  |  | - |
| *Treponema sp. HMT 951* | 1.00 | 0.00 | 0.00 | 0.00 | 0.00 |  |  | - |
| *Tannerella sp. HMT 916* | 1.00 | 0.00 | 0.00 | 0.00 | 0.00 |  |  | - |
| *Prevotella multiformis* | 1.00 | 0.00 | 0.00 | 0.00 | 0.00 |  |  | - |
| *Pyramidobacter piscolens* | 1.00 | 0.00 | 0.00 | 0.00 | 0.00 |  |  | - |
| *Treponema sp. HMT 517* | 1.00 | 0.00 | 0.00 | 0.00 | 0.00 |  |  | - |
| *Veillonellaceae [G-1] bacterium HMT 132* | 1.00 | 0.00 | 0.00 | 0.00 | 0.00 |  |  | - |
| *Corynebacterium tuberculostearicum* | 1.00 | 0.00 | 0.00 | 0.00 | 0.00 |  |  | - |
| *Prevotella sp. HMT 376* | 1.00 | 0.00 | 0.00 | 0.00 | 0.00 |  |  | - |
| *Klebsiella pneumoniae* | 1.00 | 0.00 | 0.00 | 0.00 | 0.00 |  |  | - |
| *Peptoniphilaceae [G-2] bacterium HMT 790* | 1.00 | 0.00 | 0.00 | 0.00 | 0.00 |  |  | - |
| *Treponema sp. HMT 270* | 1.00 | 0.00 | 0.00 | 0.00 | 0.00 |  |  | - |
| *Arthrospira platensis* | NA | 0.00 | 0.00 | 0.00 | 0.00 |  |  | - |
| *Lactobacillus acidophilus* | NA | 0.00 | 0.00 | 0.00 | 0.00 |  |  | - |
| *Selenomonas infelix* | NA | 0.00 | 0.00 | 0.00 | 0.00 |  |  | - |
| *Corynebacterium mastitidis* | NA | 0.00 | 0.00 | 0.00 | 0.00 |  |  | - |
| *Prevotella sp. HMT 443* | NA | 0.00 | 0.00 | 0.00 | 0.00 |  |  | - |
| *Sneathia sanguinegens* | NA | 0.00 | 0.00 | 0.00 | 0.00 |  |  | - |
| *Parvimonas sp. HMT 393* | NA | 0.00 | 0.00 | 0.00 | 0.00 |  |  | - |
| *Peptostreptococcaceae [XI][G-5] bacterium HMT 493* | NA | 0.00 | 0.00 | 0.00 | 0.00 |  |  | - |
| *Desulfovibrio fairfieldensis* | NA | 0.00 | 0.00 | 0.00 | 0.00 |  |  | - |
| *Acinetobacter junii* | NA | 0.00 | 0.00 | 0.00 | 0.00 |  |  | - |
| *Treponema sp. HMT 254* | NA | 0.00 | 0.00 | 0.00 | 0.00 |  |  | - |
| *Neisseria polysaccharea* | NA | 0.00 | 0.00 | 0.00 | 0.00 |  |  | - |
| *Peptostreptococcus anaerobius* | NA | 0.00 | 0.00 | 0.00 | 0.00 |  |  | - |
| *Lactobacillus panis* | NA | 0.00 | 0.00 | 0.00 | 0.00 |  |  | - |
| *Fretibacterium sp. HMT 358* | NA | 0.00 | 0.00 | 0.00 | 0.00 |  |  | - |
| *Selenomonas sp. HMT 479* | NA | 0.00 | 0.00 | 0.00 | 0.00 |  |  | - |
| *Peptococcus sp. HMT 168* | NA | 0.00 | 0.00 | 0.00 | 0.00 |  |  | - |
| *Streptococcus lactarius* | NA | 0.00 | 0.00 | 0.00 | 0.00 |  |  | - |
| *Simonsiella muelleri* | NA | 0.00 | 0.00 | 0.00 | 0.00 |  |  | - |
| *Bergeyella sp. HMT 422* | NA | 0.00 | 0.00 | 0.00 | 0.00 |  |  | - |
| *Anaerococcus tetradius* | NA | 0.00 | 0.00 | 0.00 | 0.00 |  |  | - |
| *Comamonas testosteroni* | NA | 0.00 | 0.00 | 0.00 | 0.00 |  |  | - |
| *Lactobacillus paracasei* | NA | 0.00 | 0.00 | 0.00 | 0.00 |  |  | - |
| *Neisseria sp. HMT 499* | NA | 0.00 | 0.00 | 0.00 | 0.00 |  |  | - |
| *Haemophilus aegyptius* | NA | 0.00 | 0.00 | 0.00 | 0.00 |  |  | - |
| *Saccharibacteria (TM7) [G-1] bacterium HMT 488* | NA | 0.00 | 0.00 | 0.00 | 0.00 |  |  | - |
| *Leptotrichia sp. HMT 879* | NA | 0.00 | 0.00 | 0.00 | 0.00 |  |  | - |
| *Bacteroides zoogleoformans* | NA | 0.00 | 0.00 | 0.00 | 0.00 |  |  | - |
| *Lactobacillus pentosus* | NA | 0.00 | 0.00 | 0.00 | 0.00 |  |  | - |
| *Lachnospiraceae [G-10] bacterium HMT 094* | NA | 0.00 | 0.00 | 0.00 | 0.00 |  |  | - |
| *Lactobacillus coleohominis* | NA | 0.00 | 0.00 | 0.00 | 0.00 |  |  | - |
| *Bacillus clausii* | NA | 0.00 | 0.00 | 0.00 | 0.00 |  |  | - |
| *Atopobium vaginae* | NA | 0.00 | 0.00 | 0.00 | 0.00 |  |  | - |
| *Propionibacteriaceae [G-1] bacterium HMT 915* | NA | 0.00 | 0.00 | 0.00 | 0.00 |  |  | - |
| *Ruminococcaceae [G-3] bacterium HMT 381* | NA | 0.00 | 0.00 | 0.00 | 0.00 |  |  | - |
| *Bergeyella sp. HMT 907* | NA | 0.00 | 0.00 | 0.00 | 0.00 |  |  | - |
| *Treponema sp. HMT 247* | NA | 0.00 | 0.00 | 0.00 | 0.00 |  |  | - |
| *Corynebacterium tuscaniense* | NA | 0.00 | 0.00 | 0.00 | 0.00 |  |  | - |
| *Lachnoanaerobaculum sp. HMT 496* | NA | 0.00 | 0.00 | 0.00 | 0.00 |  |  | - |
| *Bacteroidetes [G-3] bacterium HMT 281* | NA | 0.00 | 0.00 | 0.00 | 0.00 |  |  | - |
| *Lactobacillus rhamnosus* | NA | 0.00 | 0.00 | 0.00 | 0.00 |  |  | - |
| *Corynebacterium bovis* | NA | 0.00 | 0.00 | 0.00 | 0.00 |  |  | - |
| *Treponema sp. HMT 238* | NA | 0.00 | 0.00 | 0.00 | 0.00 |  |  | - |
| *Selenomonas sp. HMT 133* | NA | 0.00 | 0.00 | 0.00 | 0.00 |  |  | - |
| *Centipeda periodontii* | NA | 0.00 | 0.00 | 0.00 | 0.00 |  |  | - |
| *Corynebacterium pilbarense* | NA | 0.00 | 0.00 | 0.00 | 0.00 |  |  | - |
| *Corynebacterium diphtheriae* | NA | 0.00 | 0.00 | 0.00 | 0.00 |  |  | - |
| *Peptostreptococcaceae [XI][G-3] bacterium HMT 950* | NA | 0.00 | 0.00 | 0.00 | 0.00 |  |  | - |
| *Dialister sp. HMT 119* | NA | 0.00 | 0.00 | 0.00 | 0.00 |  |  | - |
| *Acinetobacter johnsonii* | NA | 0.00 | 0.00 | 0.00 | 0.00 |  |  | - |
| *Treponema sp. HMT 508* | NA | 0.00 | 0.00 | 0.00 | 0.00 |  |  | - |
| *Lactobacillus fermentum* | NA | 0.00 | 0.00 | 0.00 | 0.00 |  |  | - |
| *Treponema sp. HMT 252* | NA | 0.00 | 0.00 | 0.00 | 0.00 |  |  | - |
| *Selenomonas sp. HMT 146* | NA | 0.00 | 0.00 | 0.00 | 0.00 |  |  | - |
| *Treponema sp. HMT 249* | NA | 0.00 | 0.00 | 0.00 | 0.00 |  |  | - |
| *Treponema sp. HMT 258* | NA | 0.00 | 0.00 | 0.00 | 0.00 |  |  | - |
| *Cupriavidus gilardii* | NA | 0.00 | 0.00 | 0.00 | 0.00 |  |  | - |
| *Treponema sp. HMT 228* | NA | 0.00 | 0.00 | 0.00 | 0.00 |  |  | - |
| *Lactobacillus vaginalis* | NA | 0.00 | 0.00 | 0.00 | 0.00 |  |  | - |
| *Mycoplasma fermentans* | NA | 0.00 | 0.00 | 0.00 | 0.00 |  |  | - |
| *Haemophilus sp. HMT 259* | NA | 0.00 | 0.00 | 0.00 | 0.00 |  |  | - |
| *Moraxella osloensis* | NA | 0.00 | 0.00 | 0.00 | 0.00 |  |  | - |

Wilcoxon’s rank-sum test, *P* < 0.01

^*^ LDA score > 2 and *P* < 0.05, as determined by the linear discriminant analysis effect size (LEfSe).

**Supplementary Table S2**. Correlation of all metabolites in mouth-rinsed water with groups of bacteria significantly more prevalent in the periodontal disease group.

| **Metabolites** | **Correlation** |
| --- | --- |
| Ala | 0.69*** |
| Thr | 0.65*** |
| Tyr | 0.64*** |
| Ile | 0.63*** |
| Taurine | 0.60*** |
| Ser | 0.59*** |
| Glu | 0.59*** |
| Phe | 0.58*** |
| Val | 0.58*** |
| N-gamma-Ethylglutamine | 0.57*** |
| His | 0.57*** |
| Gln | 0.56*** |
| 5-Oxoproline | 0.56*** |
| Leu | 0.56*** |
| 2-Hydroxy-4-methylpentanoate | 0.51*** |
| Ala-Ala | 0.5*** |
| Citrulline | 0.5*** |
| N1,N12-Diacetylspermine | 0.50*** |
| Propionate | 0.48** |
| UDP-glucose | 0.48** |
| 2AB | 0.48** |
| Lys | 0.46** |
| Succinate | 0.45* |
| 2-Hydroxypentanoate | 0.44* |
| Pyruvate | 0.44* |
| 2-Hydroxyglutarate + Citramalate | 0.43* |
| Trehalose 6-phosphate | 0.42* |
| Urocanate | 0.42* |
| G6P | 0.41* |
| Homoserine | 0.41* |
| alpha-Aminoadipate | 0.41* |
| Gly | 0.41* |
| Asp | 0.40* |
| Hypoxanthine | 0.39* |
| 3'-AMP | 0.39* |
| GABA | 0.38* |
| Nicotinate | 0.38* |
| F6P | 0.37* |
| AMP | 0.37* |
| N-Acetylglutamate | 0.37* |
| Cadaverine | 0.36* |
| Cysteine-glutathione disulphide | 0.36* |
| N-Acetyl-beta-alanine | 0.36 |
| Threonate | 0.36 |
| UMP | 0.35 |
| beta-Ala-Lys | 0.35 |
| Glutathione(ox) | 0.34 |
| N-Acetylornithine | 0.33 |
| Inosine | 0.32 |
| Trigonelline | 0.31 |
| 3PG + 2PG | 0.31 |
| Thymine | 0.31 |
| o-Hydroxybenzoate | 0.31 |
| Carnitine | 0.30 |
| PEP | 0.30 |
| 6-Phosphogluconate | 0.30 |
| Adenine | 0.30 |
| Butyrate | 0.29 |
| 3-Phenyllactate | 0.28 |
| Sarcosine | 0.27 |
| 4-Methyl-2-oxopentanoate | 0.27 |
| Glycerophosphorylcholine | 0.26 |
| Malate | 0.26 |
| Choline | 0.25 |
| Agmatine | 0.25 |
| Trp | 0.25 |
| Arg | 0.25 |
| Guanine | 0.24 |
| beta-Ala | 0.24 |
| 3-(4-Hydroxyphenyl)propionate | 0.24 |
| 1-Methyl-2-pyrrolidinone | 0.24 |
| Ornithine | 0.24 |
| CMP | 0.23 |
| Homovanillate | 0.23 |
| N-Acetylglucosamine 6-phosphate | 0.23 |
| Benzamide | 0.23 |
| Fumarate | 0.22 |
| Quinate | 0.22 |
| 3-Methylhistidine | 0.21 |
| Lactate | 0.21 |
| DHAP | 0.21 |
| S7P | 0.20 |
| Pro | 0.20 |
| Met | 0.20 |
| Guanosine | 0.20 |
| Spermine | 0.20 |
| Glu-Glu | 0.20 |
| N-Acetylputrescine | 0.18 |
| Pipecolate | 0.17 |
| Phe-Phe | 0.17 |
| Pentanoate + 3-Methylbutanoate | 0.17 |
| Hydroxyproline | 0.17 |
| Spermidine | 0.17 |
| gamma-Butyrobetaine | 0.17 |
| 3-Hydroxypropionate | 0.16 |
| 7-Methylguanine | 0.16 |
| Creatine | 0.14 |
| G1P | 0.14 |
| 3-Aminoisobutyrate | 0.12 |
| Histamine | 0.12 |
| Glucosamine | 0.12 |
| 2-Oxoisopentanoate | 0.12 |
| 2-Hydroxybutyrate | 0.12 |
| R5P | 0.11 |
| 5-Aminolevulinate | 0.10 |
| Indole-3-acetate | 0.10 |
| Ru5P | 0.10 |
| Putrescine(1,4-Butanediamine) | 0.10 |
| SAM+ | 0.10 |
| 3-Hydroxybutyrate | 0.09 |
| o-Acetylcarnitine | 0.08 |
| Thymidine | 0.08 |
| Isocitrate | 0.07 |
| 5-Methoxyindoleacetate | 0.06 |
| Asn | 0.06 |
| Malonate | 0.06 |
| cis-Aconitate | 0.05 |
| Isopropanolamine | 0.05 |
| Gly-Gly | 0.01 |
| 2-Isopropylmalate | 0.00 |
| Hexanoate | 0.00 |
| Nicotinamide | 0.00 |
| Tyramine | 0.00 |
| Cytidine | -0.02 |
| 10-Hydroxydecanoate | -0.02 |
| Nicotine | -0.02 |
| N-Acetylglucosamine 1-phosphate | -0.03 |
| Glycolate | -0.04 |
| Betaine | -0.04 |
| Allantoin | -0.04 |
| Citrate | -0.06 |
| Phthalate | -0.08 |
| 5-Aminovalerate | -0.08 |
| Dodecanedioate | -0.11 |
| Phosphorylcholine | -0.13 |
| Indole-3-acetaldehyde | -0.13 |
| Carnosine | -0.13 |
| N-Acetylneuraminate | -0.14 |
| Azelate | -0.14 |
| Diethanolamine | -0.14 |
| Creatinine | -0.14 |
| Sebacate | -0.15 |
| Glycerophosphate | -0.16 |
| Urate | -0.17 |
| Trimethylamine N-oxide | -0.18 |
| Uridine | -0.18 |
| 3-Phenylpropionate | -0.18 |
| Anserine + Homocarnosine | -0.21 |
| Adenosine | -0.21 |
| N-Acetylaspartate | -0.21 |
| Riboflavin | -0.24 |
| 1,3-Diaminopropane | -0.27 |
| N-Acetylglucosamine | -0.30 |
| Ethanolamine phosphate | -0.30 |
| Urea | -0.52 |

Spearman’s correlation analysis was performed, and significant differences were calculated using the Bonferroni method. A Spearman’s rank correlation coefficient of 0.4 indicated correlation, and the two-sided significance level was set at 5%. Bonferroni method *P* < 0.05.

**Supplementary Table S3**. GC-MS/MS conditions.

|  | | Propionate | Homoserine | 5-Oxoproline |
| --- | --- | --- | --- | --- |
| Pretreatment method | | *t*-BDMS Derivatization | TMS Derivatization | |
| column | | DB-5 (I.D. = 0.25 mm, L = 30 m, df = 1.0 µm, Agilent Technologies) | DB-5 (I.D. = 0.25 mm, L = 30 m, df = 1.0 µm, Agilent Technologies) | |
| Column Temp. | | 60℃, 3 min → (10℃/min) → 100℃ → (20℃/min) → 200℃ → (40℃/min) → 310℃, 5 min | 100℃, 4 min → (10℃/min) → 230℃ → (40℃/min) → 320℃, 5 min | |
| Inlet temp. | | 150℃, 0.5 min → (25℃/min) → 290℃, 13.65 min | 220℃, 0.5 min → (50℃/min) → 290℃, 22.35 min | |
| Injection mode | | Split (20:1) | Split (20:1) | |
| Injection volume | | 30 µL | 27 µL | |
| control mode | | linear velocity | linear velocity | |
| carrier gas | | Helium | Helium | |
| linear velocity | | 39.0 cm/s | 39.0 cm/s | |
| Column Flow Rate | | 1.14 mL/min | 1.10 mL/min | |
| Purge flow rate | | 5.0 mL/min | 5.0 mL/min | |
| Interface Temp. | | 290℃ | 280℃ | |
| Ion Source Temp. | | 200℃ | 200℃ | |
| Ionizing voltage | | 70 eV | 70 eV | |
| Ionizing mode | | EI | EI | |
| Measurement Mode | | MRM | MRM | |
| MRM Conditions | Quantity | Q1 (precursor, m/z):131.00 > Q3 (product, m/z):75.10, Collision Energy: 15.00 V | Q1 (precursor, m/z): 218.00 > Q3 (product, m/z): 73.10, Collision Energy: 24.00 V | Q1 (precursor, m/z): 156.00 > Q3 (product, m/z): 73.10, Collision Energy: 15.00 V |
|  | Quality | Q1 (precursor, m/z): 75.00 > Q3 (product, m/z): 60.10, Collision Energy: 15.00 V | Q1 (precursor, m/z): 218.00 > Q3 (product, m/z): 128.10, Collision Energy: 9.00 V | Q1 (precursor, m/z) 147.00 > Q3 (product, m/z): 73.10, Collision Energy: 18.00 V |
|  | Quality | Q1 (precursor, m/z): 131.00 > Q3 (product, m/z): 60.10, Collision Energy: 27.00 V | Q1 (precursor, m/z): 218.00 > Q3 (product, m/z): 73.10, Collision Energy: 12.00 V | Q1 (precursor, m/z): 73.00 > Q3 (product, m/z): 58.10, Collision Energy: 18.00 V |

**Supplementary Table S4**. Optimized MRM and PRM parameters for each metabolite and its isotopomer.

|  | **Q1** | **Q3** | **Fragmentor (v)** | **Collision Energy (v)** | **Polarity** |
| --- | --- | --- | --- | --- | --- |
| ***N*1, *N*12-Diacetylspermine** | 287 | 100 | 125 | 24 | Positive |
| ***N*1, *N*12-Diacetylspermine-d6** | 293 | 103 | 125 | 24 | Positive |
| **Citrulline** | 176.1 | 159 | 90 | 5 | Positive |
| **Citrulline-13C5** | 181.2 | 164.1 | 90 | 9 | Positive |
| **Succinate** | 117.0 | 73.02 | - | 35 | Negative |
| **Succinate-d6** | 123.0 | 77.05 | - | 35 | Negative |

**Supplementary Methods S1**

*Gas chromatograph-mass spectrometry (GC-MS/MS)*

The harvested bacterial culture solution was diluted 50-fold to a total volume of 200 µL using Milli-Q water, after which 800 µL of acetonitrile was added. Centrifugation (MDX-310; TOMY SEIKO Co., Ltd., Tokyo, Japan) was conducted at 17,750 × *g* and 4℃ for 3 min, and 2.5 μL of 1 N NaOH was added to 800 μL of supernatant for derivatization. Based on previous reports^1^ ^2^, derivatization was performed using an SGI-M100 online solid-phase extraction-gas chromatography system (AiSTI SCIENCE Co., Ltd., Wakayama, Japan). Propionate was converted into *tert*-butylchlorodimethylsilane (*t*-BDMS) derivatives using a Flash-SPE AX solid-phase cartridge (AiSTI SCIENCE Co., Ltd.), and 5-oxoproline and homoserine were converted into trimethylsilyl (TMS) derivatives using Flash-SPE ACX (AiSTI SCIENCE Co., Ltd.) (Supplementary Figure S1). The derivatized products were injected into a GCMS-TQ8040 system (Shimadzu, Kyoto, Japan) using an LVI-S250 programmable temperature vaporization injector (AiSTI SCIENCE Co., Ltd.). The column used was a DB-5 column (I.D. = 0.25 mm, df = 1.0 um, L = 30 m; Agilent Technologies), and an LA-5010-004 spiral insert (deactivated; AiSTI SCIENCE Co., Ltd.) was used for injection. Measurements were performed according to the conditions listed in Supplementary Table S3, and GCMS solution ver 4.52 (Shimadzu) was used for data analysis.

**Supplementary Methods S2**

*Multisegment injection-CE-MS/MS (MSI-CE-MS/MS)*

The harvested bacterial culture solution was diluted 2-fold with methanol and centrifuged (MDX-310; TOMY SEIKO Co., Ltd.) at 17,750 × *g* and 4℃ for 3 min, after which 50 μL of supernatant was suspended in 130 μL of MeOH. Next, 200 μL of chloroform and 100 μL of 10 μM Citrulline-13C5 [L-CITRULLINE (1,2,3,4,5-13C5, 98%); Cambridge Isotope Laboratories, Inc., Tewksbury, MA, USA] were added, and centrifugation was conducted at 4,600 × *g* and 4℃ for 5 min. Subsequently, the aqueous phase was transferred to an ultrafiltration filter, centrifuged at 9,100 × *g* and 4℃ for approximately 3 h to remove proteins of 5 kDa or larger, and subjected to centrifugal concentration drying for approximately 3 h using a Refrigerated CentriVap Concentrator (LABCONCO, Kansas City, MO, USA). Afterward, 50 μL of Milli-Q water was added, and the mixture was subjected to MSI-CE-MS/MS, changing the following conditions with reference to the methods of Igarashi et al^3^. using a CE: Agilent 7100 Capillary Electrophoresis System (G7100A), MS/MS: Agilent 6470 LC/TQ System (G6224A), Pump: Agilent 1260 Isocratic HPLC pump (G7110B), G1603A Agilent CE-MS adapter kit, and G1607A Agilent CE-electrospray ionization (ESI)-MS sprayer kit (Agilent Technologies). The sheath fluid was a 50% methanol solution, the background electrolyte was set to 60 s, and the nebulizer gas was set to 7 psi. The measurement mode adopted was MRM, whose conditions are listed in Supplementary Table S4. Keio MSI-MasterHands ver 1.3.2 was used for data analysis.

**Supplementary Methods S3**

*Ion chromatograph-mass spectrometry (IC-MS/MS)*

The harvested bacterial culture solution was diluted 2-fold with methanol and centrifuged (MDX-310; TOMY SEIKO Co., Ltd.) at 17,750 × *g* and 4℃ for 3 min, after which 50 μL of supernatant was suspended in 130 μL of MeOH. Thereafter, 200 μL of chloroform and 100 μL of 5 µM succinate-d6 [succinic acid-d6 (99%); CDN Isotopes, Inc., Pointe-Clair, QC, Canada] were added, and centrifugation was conducted at 4,600 × *g* and 4℃ for 5 min. Next, the aqueous phase was transferred to an ultrafiltration filter, centrifuged at 9,100 × *g* and 4℃ for approximately 3 h to remove proteins of 5 kDa or larger, and subjected to centrifugal concentration drying for approximately 3 h using a Refrigerated CentriVap Concentrator (LABCONCO). Subsequently, 50 μL of Milli-Q water was added, and the mixture was subjected to IC-MS/MS using a Dionex ICS-500+ system (Thermo Fisher Scientific, Waltham, MA, USA) equipped with a Q-Exactive Plus system (Thermo Fisher Scientific). The measurement mode adopted was PRM, whose conditions are listed in Supplementary Table S4. Other analytical conditions were as previously described^4^.

**1 Yamamoto, K. *et al.* Metabolomic investigation of differences in components and taste between hon-mirin and mirin-like-seasoning. *J. Biosci. Bioeng.* 132, 599-605, doi:10.1016/j.jbiosc.2021.08.011 (2021).**

**2 Hata, S. *et al.* Gut Microbiota Changes by an SGLT2 Inhibitor, Luseogliflozin, Alters Metabolites Compared with Those in a Low Carbohydrate Diet in db/db Mice. *Nutrients* 14, doi:10.3390/nu14173531 (2022).**

**3 Igarashi, K. *et al.* High-throughput screening of salivary polyamine markers for discrimination of colorectal cancer by multisegment injection capillary electrophoresis tandem mass spectrometry. *J. Chromatogr. A* 1652, 462355, doi:10.1016/j.chroma.2021.462355 (2021).**

**4 Watanabe, M. *et al.* Extracellular N-acetylaspartylglutamate released in the nucleus accumbens modulates the pain sensation: Analysis using a microdialysis/mass spectrometry integrated system. *Mol. Pain* 14, 1744806918754934, doi:10.1177/1744806918754934 (2018).**
